# Seasonal asthma in Melbourne, Australia, and some observations on the occurrence of thunderstorm asthma and its predictability

**DOI:** 10.1371/journal.pone.0194929

**Published:** 2018-04-12

**Authors:** Jeremy D. Silver, Michael F. Sutherland, Fay H. Johnston, Edwin R. Lampugnani, Michael A. McCarthy, Stephanie J. Jacobs, Alexandre B. Pezza, Edward J. Newbigin

**Affiliations:** 1 School of Earth Sciences, University of Melbourne, Parkville, Victoria, Australia; 2 Institute of Breathing and Sleep, Department of Medicine, University of Melbourne, Parkville, Victoria, Australia; 3 Austin Health, Heidelberg, Victoria, Australia; 4 Menzies Institute for Medical Research, University of Tasmania, Hobart, Tasmania, Australia; 5 School of BioSciences, University of Melbourne, Parkville, Victoria, Australia; 6 School of Earth, Atmosphere & Environment, Monash University, Clayton, Victoria, Australia; 7 Greater Wellington Regional Council, Pipitea, Wellington, New Zealand; 8 School of Geography, Environment and Earth Sciences, Victoria University of Wellington, Wellington, New Zealand; National Sun Yat-sen University, TAIWAN

## Abstract

We examine the seasonality of asthma-related hospital admissions in Melbourne, Australia, in particular the contribution and predictability of episodic thunderstorm asthma. Using a time-series ecological approach based on asthma admissions to Melbourne metropolitan hospitals, we identified seasonal peaks in asthma admissions that were centred in late February, June and mid-November. These peaks were most likely due to the return to school, winter viral infections and seasonal allergies, respectively. We performed non-linear statistical regression to predict daily admission rates as functions of the seasonal cycle, weather conditions, reported thunderstorms, pollen counts and air quality. Important predictor variables were the seasonal cycle and mean relative humidity in the preceding two weeks, with higher humidity associated with higher asthma admissions. Although various attempts were made to model asthma admissions, none of the models explained substantially more variation above that associated with the annual cycle. We also identified a list of high asthma admissions days (HAADs). Most HAADs fell in the late-February return-to-school peak and the November allergy peak, with the latter containing the greatest number of daily admissions. Many HAADs in the spring allergy peak may represent episodes of thunderstorm asthma, as they were associated with rainfall, thunderstorms, high ambient grass pollen levels and high humidity, a finding that suggests thunderstorm asthma is a recurrent phenomenon in Melbourne that occurs roughly once per five years. The rarity of thunderstorm asthma events makes prediction challenging, underscoring the importance of maintaining high standards of asthma management, both for patients and health professionals, especially during late spring and early summer.

## Introduction

Seasonal variation in admissions to hospital for the treatment of asthma is a phenomenon that has been documented in many different regions of the world [1, 2]. Previous studies have found associations between asthma exacerbations and both higher and lower air temperatures or humidity [3], the change in air temperature within a day [4], the prevalence of respiratory viral infections [5], and outdoor concentrations of air pollutants and allergens [6]. A well-recognised pattern is the autumn peak of asthma exacerbations in school children associated with returning to school in February in the Southern hemisphere, and September in the northern hemisphere [7, 8]. Individual clinical characteristics (e.g. impaired pulmonary function) also explain a large proportion of the variation in seasonal and geographic patterns in asthma [9, 10].

Thunderstorm asthma is a less well-recognised seasonal syndrome that is characterised by a sudden increase in the number of people with symptoms of acute allergic asthma following a thunderstorm [11]. Apart from a thunderstorm, high levels of airborne allergens such as pollen grains and fungal spores are thought necessary. Severe episodes of thunderstorm asthma, when there is a large spike in hospital admissions, can rapidly overwhelm local health services and place hospital and intensive care units under strain. In Australia, documented episodes of thunderstorm asthma have all occurred in late spring or early summer, when the grass pollen season and increased thunderstorm activity coincide [12, 13]. However, the mechanisms by which thunderstorms and aeroallergens interact with individual risk factors to cause severe allergic asthma are only partly understood. A commonly cited explanation is that changes in pressure, temperature or humidly associated with thunderstorms cause the rupture of whole pollen grains, releasing small allergenic fragments that are able to travel beyond the pharynx to the small airways, with cold down-drafts or outflows from the thunderstorm helping to concentrate these allergenic fragments at ground-level [12, 14]. However, not all thunderstorms are associated with the phenomenon, severe episodes are relatively uncommon [11], and a range of allergenic particles including fungi and pollen have all been implicated [15].

Thunderstorm warnings have been issued by meteorological agencies for decades [16] and operational pollen forecasts exist for certain taxa in some parts of Europe, North America and Australia [17, 18]. Even though in regions where thunderstorm asthma occurs, it is acknowledged that a better understanding of the contributing factors would assist local health services in planning and asthmatic individuals in managing their condition. We are only aware of two thunderstorm asthma prediction services, namely those operating in Wagga Wagga (in New South Wales) [19] and Victoria [20]. Melbourne, Australia, is a particularly interesting location in this regard, as between 1984 and 2016 the city experienced at least six thunderstorm asthma events, with the last of these events on 21^st^ November 2016, being the most extreme [13, 21–25].

Here we aim to evaluate the potential for predicting thunderstorm asthma episodes by studying asthma-related hospital admissions in Melbourne over a 16-year period (n.b. this period does not include the event on November 21, 2016 as admissions data for the year were not available). We begin by examining the seasonality of asthma for the whole population and different age groups, and use non-linear statistical regression to predict the daily numbers of asthma-related admission. The predictor variables in these regression analyses account for seasonal variation, thunderstorm indicators, weather, air quality and ambient pollen concentrations. We examine the association between these environmental factors and asthma admission rates. From this time-series, we identify days with unusually high asthma admissions and test the capability of our models for predicting these high asthma admission days.

## Materials and methods

### Data

#### Health records

Admissions data for public hospitals within the Greater Melbourne region for the period January 1990 to June 2015 were provided by the Victorian Department of Health and Human Services and were extracted from the Victorian Admitted Episodes Dataset. Diagnoses were coded according to the World Health Organisation’s International Classification of Diseases (ICD) 9^th^ revision (ICD-9) until March 1998, and thereafter according to the 10^th^ revision (ICD-10). Data were extracted for patients with a principal diagnosis of asthma (ICD-9 code 493 and ICD-10 codes J45, J46) and included the patient’s age (by 5-year intervals: 0-4, 5-9, …, 80-84 and 85+), gender and the local government region of patient’s place of residence. Time of admission was not provided, so the number per day spans 00:00h until 23:59h. Data were available for 9312 days and 170344 admissions in total, with a mean admission rate of 0.543 admissions per day per 100,000 population (all age groups included). The project was approved by the Human Research Ethics Committee of Austin Health.

#### Airborne pollen

Daily pollen concentrations were measured from October 1^st^ to December 31^st^ each year since 1991, a period that includes the grass pollen season in Melbourne [18]. The sampling site at the University of Melbourne (in Parkville, Victoria) is 1.7 km north of central Melbourne (defined as the General Post Office). Measurements were taken at 13 m above ground level until 2006 and subsequently at a nearby site 20 m above ground level. Sampling locations are shown in S1 Fig. This dataset spans most of the period of the asthma admission records. Samples were collected using a Burkard volumetric trap (Burkard Manufacturing Co. Ltd., Rickmansworth, Hertfordshire, UK), and pollen grains were identified by microscopy (for protocols, see [26]). The classification was limited to grass taxa or non-grass taxa. The choice to focus on grass pollen (as opposed to other aeroallergens) was based on the availability of this data and the assumption that grass was the major outdoor aeroallergen in the region, a finding supported by previous studies [23]. Measured concentrations represent a 24-hour average from 16:00h one day to 16:00h the next day. The measured concentration is associated with the day in which the measurement concluded (as this day covers 67% of the measurement period). While the grass pollen season in the Melbourne region peaks around November and December, pollen will be present in the air throughout the year, with several tree species emitting large quantities of pollen in the months of July through to September [26]. However, data on other taxa were not available for the time-period of this study.

#### Meteorology

We used half-hourly measurements of wind speed and direction, precipitation, temperature and relative humidity; these data were recorded by the Australian Bureau of Meteorology at Melbourne Airport, located 19 km to the north north west of central Melbourne (S1C Fig). Values from midday were used in the regression analyses, apart from precipitation (which used total daily rainfall from 00:00h until 23:59h). The choice of the midday snapshot, rather the daily averages, was related to the assumption that day-time values were more representative of exposure than averages; furthermore, in the case of allergic asthma, aeroallergens such as pollen can be assumed to be found at higher concentrations during the day (due to stronger wind speeds and greater surface heating). The analysis presented below was performed using either midday meteorological snapshots or daily mean values, and while regression coefficients showed minor differences the resulting conclusions were ostensibly identical.

For the identification of thunderstorms, several different thunderstorm indicators were considered (discussed in detail in Appendix 1). A thunderstorm was deemed to have occurred when reported by the meteorological observers at Melbourne Airport; this corresponded to the code “TS” in the aviation weather reports (METAR/SPECI). This site has a wide field of view and is staffed continuously by trained meteorological observers, hence it provides an excellent vantage point for detection of thunderstorm activity in the vicinity of the city.

#### Air quality

Hourly measurements of ozone (O_3_) and airborne particulate matter with an aerodynamic diameter less than 2.5 *μ* m (PM_2.5_) or 10 *μ* m (PM_10_) were measured by the Victorian Environmental Protection Agency. Data were available at a total of nine sites (not all species were recorded at each site). The monitoring network changed over the 11 years that these data spanned, and as such we chose to use only data from a single monitoring site (in Alphington, 7.1 km north east of the Melbourne central business district) rather than taking a spatial average. The Alphington monitoring site was the closest long-term monitoring site to the city not affected by major industrial developments. For each species, we calculated average concentrations across all hours of the day (i.e. 00:00h until 23:59h). These data were available only for the period 2003-2014. In the analysis presented below, we did not use PM_10_ as an explanatory variable, as it is highly correlated with PM_2.5_ (*R*^2^ > 0.74), and of the two PM_2.5_ is associated with stronger health effects [27].

### Analysis

#### Analysing patterns of temporal variation

**Fig 1:**
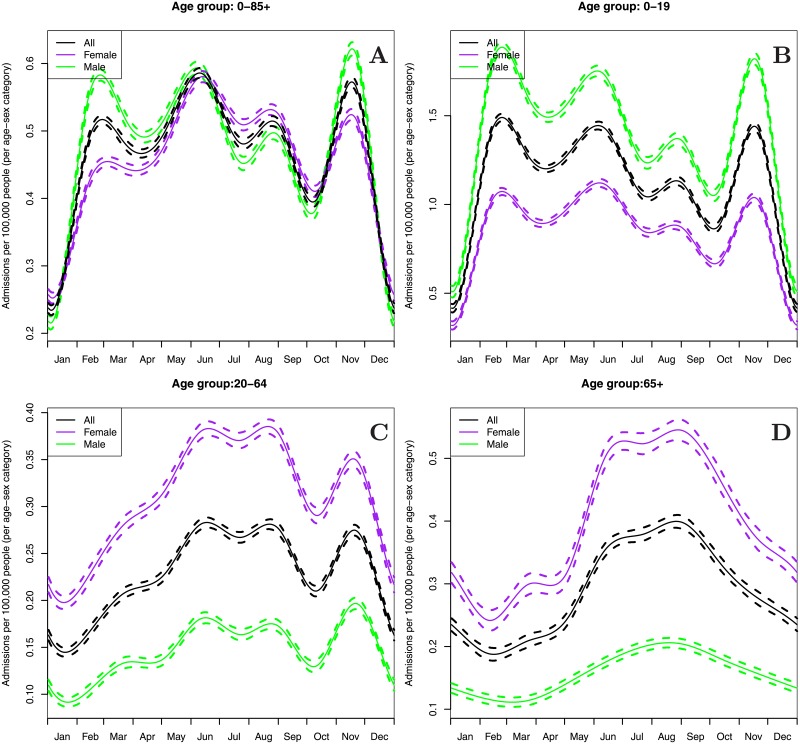
seasonal cycle in population-normalised asthma-related hospital admissions. The seasonal cycle in population-normalised asthma-related hospital admissions, normalised by the population size in each age-gender category. The individual panels show the cycle for the full population (A), children and teenagers (B), working-aged adults (C) and retiree-aged adults (D). The dashed lines show the effect plus or minus one standard error of the fitted cyclical cubic spline. Note the different scales on the *y*-axis in the four panels.

The full time-series of asthma admissions (1990-2015) exhibited variation on a range of scales. A strong annual cycle was observed, as well as a trend in raw admissions (S3 Fig). The trend can partly be understood by growth of 42% between 1991 and 2015 in Melbourne’s population [28, 29]; however, even population-normalised asthma admissions showed considerable heteroscedasticity (S4 Fig). In order to estimate the annual cycle of asthma admission rates, we fitted a smooth curve (a cyclical cubic spline) to the population-adjusted daily admission rate as a function of day-of-year. This was done both for all individuals in the dataset and separately for different age groups (Fig 1). For the spline fit, the number of degrees of freedom was estimated by generalised cross validation [30].

#### Prediction of asthma hospital presentation rates via regression

We used non-linear statistical regression to help understand factors affecting day-to-day variation in asthma admissions, and assess the predictability of such admissions. When configuring the regression models, various choices had to be made as to which variables to include, how to treat these variables and the time period to be considered. As these choices affected the results, we shall present the outcomes from eight different regression models (summarised in S2 and S3 Tables) and describe findings that were consistent across multiple models.

Generalized additive models, or GAMs [30, 31], were fitted to the population-adjusted daily admission rate, with the following predictor variables: the day-of-year (as a continuous variable), the day of the week (as a categorical model), relative humidity, temperature, precipitation, north-south wind speed, east-west wind speed, thunderstorms, grass pollen concentration and non-grass pollen concentration. The GAMs allowed for non-linear relationships between the dependent and the continuous independent variables (S6 to S13 Figs). Additional GAMs were fitted to examine the effect of air pollutants (O_3_ and PM_2.5_) and the interaction between thunderstorms and pollen. Further, the temperature and relative humidity terms were decomposed into two components: a rolling, backward-looking 14-day mean and the daily deviation from this rolling mean (the sum of the two components is the original daily value). Similarly, in some of the regression models the mean pollen concentration for the previous 3 days were included as predictor variables; these “lagged” means did not include the value from the day of interest. Automatic smoothness selection was applied using generalised cross validation, aiming to find a balance between additional degrees of freedom and minimising the out-of-sample prediction error (similar to minimising the Akaike Information Content); this process allowed for implicit variable selection, such that terms that did not appear to contribute to the fit were effectively dropped from the model. S6 to S13 Figs show the fitted splines from the GAMs.

The full time-series (1990-2015) could not be used because, despite careful matching of the ICD-9 and ICD-10 codes, inconsistencies between the two coding periods were observed. Furthermore, a change occurred in 1999 in the archiving of meteorological observer data from Melbourne Airport that affected the consistency of the thunderstorm indicator used in this study. Accordingly, regression analysis was performed for the period 2000-2015. When air quality variables were included, data availability further restricted the period being considered to 2003-2014. Models using meteorological and air quality variables only were fitted for the full year, and models also using the pollen data were fitted for the October to December period due to the limited availability of these data (see S3 Table).

The results of the regression models are presented as *t*-statistics, *F*-statistics and *p*-values from the associated tests of the model parameters, as well as an estimate of the “effect size” (S4 to S11 Tables, Appendix 2). The effect size aims to quantify the practical significance (c.f. statistical significance) of the variable. The effect sizes reported are an estimate of the number of asthma admissions in a population of 4 million (n.b. the population of Melbourne grew from to 3.45 to 4.53 million over the period June 2000 to June 2015 [28, 29]) attributed to this explanatory variable (e.g. precipitation) when this variable is at its 95% quantile (or, in the case of binary or categorical variables, simply when it applies).

#### Prediction of high-asthma episodes

In order to identify high asthma admissions days (HAADs) relative to the admissions in the surrounding period, we began by calculating normalised residuals from the admissions time-series. These were obtained by subtracting from the original admissions time-series a running trimmed mean and then dividing the result by a running trimmed standard deviation; both trimmed statistics were based only on the central 50% of values within the centred 31-day window (S5 Fig). We defined a HAAD as having a normalised residual of 4.5 or greater (i.e. 4.5 local standard deviations above the local mean), as this corresponded to a sharp departure from an otherwise smooth curve in the Gaussian quantile-quantile plot (S14 Fig). A total of 16 HAADs met this criterion, of which 10 were in the October-December period, one was in mid-September, and five fell in mid- to late-February (Table 1, S15 Fig). A logistic regression model was used to predict the binary variable of whether a given day would be a HAAD or not; the choice of predictor variables was the similar to model 3 (S3 Table) but with two exceptions. Firstly, spline terms were replaced with linear terms (apart the day-of-year effect, for which the fitted term shown in Fig 1A was used), and secondly the weekday effect was replaced by a binary working day vs. non-working day (accounting for both weekends and public holidays). Both of these measures were required to reduce the number of degrees of freedom of the model, given the small number of HAADs available to fit such models. Only the October-December period was used to fit this model (given the use of the pollen variables). A random sample of 70% of individual days was used to train the model, while the remaining 30% was reserved to evaluate the skill of the predictive model. This predictive process was repeated on 100 randomly sampled test and training sets. The number of HAADs varied in each random sample, but at least two HAADs were present in each test set (if not, the random selection was performed again).

**Table 1:** Details of the HAADs. Abbreviations: WK = day of week, RA = raw admission numbers, NA = normalised admissions given as the number of admissions per 100,000 population, WS = wind speed at Melbourne airport at midday (units = km/h), WD = wind direction (i.e. from which the wind is blowing) at Melbourne airport at midday (units = degrees clockwise from North), EW = east-west component of the wind-speed at Melbourne airport at midday (units = km/h, positive means winds from the west), NS = north-south component of the wind-speed at Melbourne airport at midday (units = km/h, positive means winds from the south), PR = precipitation at Melbourne airport from 00:00h to 23:59h (units = mm), TM = temperature at Melbourne airport at midday (units = ° C), RH = relative humidity at Melbourne airport at midday (units = %), TS = thunderstorm reported at Melbourne airport from 00:00h to 23:59h (Y = yes, N = no), GR = daily grass pollen concentration at the University of Melbourne averaged from 16:00h the previous day to 16:00h the date stated (units = grains/m^3^), NG = daily non-grass pollen concentration, *x*_lg_ = “lagged” average value of variable *x* over the 3 days prior to the given day (i.e. not including the value on the given day), O_3_ = daily average ozone (units = parts per billion by volume), PM_2.5_ = daily average particulate matter with an aerodynamic diameter less than 2.5 *μ* m (units = *μ* g/m^3^), NA = not available.

Date	WK	RA	NA	WS	WD	EW	NS	PR	TM	RH
1993-02-14	Su	69	2.3	28	360	0.0	-28.0	0.0	27	37
1993-02-15	Mo	70	2.3	59	350	10.2	-58.1	0.8	24	47
1994-11-20	Su	52	1.7	26	310	19.9	-16.7	3.6	23	33
1996-11-03	Su	52	1.7	37	360	0.0	-37.0	2.5	19	73
2001-02-18	Su	54	1.7	42	10	-7.3	-41.4	0.0	29	28
2001-11-25	Su	53	1.6	31	340	10.6	-29.1	1.2	20	38
2003-11-20	Th	76	2.3	22	350	3.8	-21.7	4.4	30	45
2005-02-21	Mo	47	1.4	13	250	12.2	4.4	0.0	22	67
2009-02-16	Mo	52	1.4	9	70	-8.5	-3.1	0.0	24	44
2009-10-31	Sa	58	1.5	26	10	-4.5	-25.6	9.4	29	39
2009-11-01	Su	49	1.3	2	90	-2.0	0.0	1.6	16	90
2010-11-13	Sa	71	1.8	21	180	0.0	21.0	19.8	14	94
2010-11-25	Th	144	3.7	24	220	15.4	18.4	23.0	19	92
2011-10-29	Sa	65	1.7	22	240	19.1	11.0	11.4	19	56
2011-11-08	Tu	73	1.9	13	70	-12.2	-4.4	7.6	22	71
2013-09-16	Mo	55	1.3	21	10	-3.6	-20.7	1.8	16	64
Date	TS	GR	NG	GR_lg_	NG_lg_	O_3_	PM_2.5_			
1993-02-14	Y	NA	NA	NA	NA	NA	NA			
1993-02-15	N	NA	NA	NA	NA	NA	NA			
1994-11-20	N	107	336	73	126	NA	NA			
1996-11-03	Y	251	281	102	258	NA	NA			
2001-02-18	N	NA	NA	NA	NA	NA	NA			
2001-11-25	N	48	151	76	227	NA	NA			
2003-11-20	Y	60	761	91	820	24	9			
2005-02-21	N	NA	NA	NA	NA	11	8			
2009-02-16	N	NA	NA	NA	NA	17	22			
2009-10-31	Y	105	126	31	84	25	5			
2009-11-01	N	22	44	59	100	18	5			
2010-11-13	N	24	130	147	492	14	NA			
2010-11-25	N	23	109	92	675	11	3			
2011-10-29	N	32	84	76	385	NA	NA			
2011-11-08	Y	98	660	109	489	28	NA			
2013-09-16	Y	NA	NA	NA	NA	NA	NA			

We also examined the predictive skill of a machine-learning classifier; the method chosen was “gradient boosting” [32], which builds a classification model from an ensemble of “base learners”. Linear base learners were used for most variables, smoothing splines were used for precipitation, rolling mean temperature and rolling mean humidity (see S6 to S13 Figs for evidence of non-linearity in the GAM fits), and a classification-tree based base-learner was used for the thunderstorm and pollen variables (thus allowing for interactions).

#### Statistical software

The R statistical computing environment was used for all analyses [33]. The R package mgcv was used for fitting the GAMs with penalised regression splines [31]. Gradient boosting was performed using the R package mboost [34].

## Results

### Seasonality

In the full time-series (1990-2015) there was a clear seasonal cycle of asthma emergency department presentations with four peaks that were centred in late February, June, August and mid-November (Fig 1A). The late February peak was likely due to the return-to-school effect as it occurred two to three weeks after the start of the school year following the summer holidays and primarily affected children and adolescents [35], with a smaller effect seen in working-aged adults (Fig 1B and 1C). Similarly, the June and August peaks (which are not differentiated in the 65+ age-group) were most likely related to the winter-time exacerbations induced by upper-respiratory viral infections [36]. The November peak, presumably related to seasonal allergies, has a similar height to the June peak in the full population and was the largest of the asthma admissions peaks for children and adolescents; it was not evident, however, in the 65+ age-group.

Most admitted individuals were children or teenagers, with 52% of admissions aged 0-9, 11% aged 10-19, 29% aged 20-64 and 8% aged 65 or older. Within each age-group, there were clear differences in the overall admission rates between males and females (Fig 1), with more male patients than female patients among children and younger teenagers (below 15), and more female than male patients among older teenagers (aged 15 or more) and adults. These patterns are well-recognised in asthma admissions [37]. Apart from the differences in magnitude, the seasonal cycle of admissions were generally very similar for male and female patients within a given age-group.

**Fig 2:**
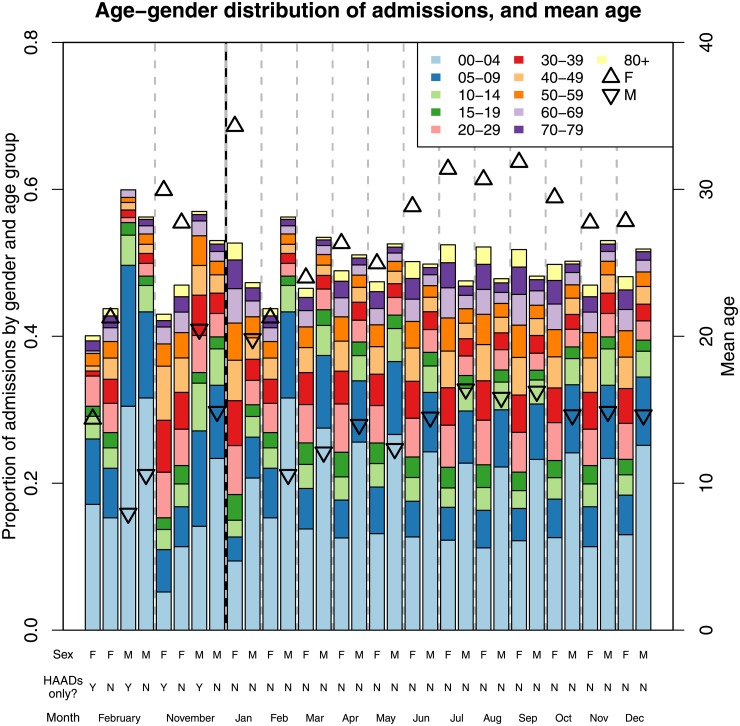
Proportion of admissions by age and gender over the year and for HAADs. The proportion of admissions by gender and five-year age-group (stacked bar-chart) and the mean age of admitted individuals per gender (triangles), shown for HAADs in February and November and each month of the year. The left *y*-axis applies to the stacked bar-charts, while right *y*-axis applies to the points. The *x*-axis labels show the set of days and the gender of the individuals. To the left of the vertical black-grey dashed line is the same representation of the age-gender distribution for HAADs only or all days. Vertical grey dashed lines separate data for different months.

Closer examination of the distribution of admissions by age and sex (Fig 2) shows that the largest group of asthma patients was comprised of boys aged less than 10, constituting 30-40% of admissions in most months, and around 50% for HAADs in February. Girls aged less than 10 represented 20% or less of all admissions in most months except February. The mean age of male patients was around 10-15 years, while the mean age of female asthma patients admitted to hospital ranged between 20-33 years. Male admissions were more common in all months other than January (when there were very few admissions) and from June to August (coinciding with the winter peak). While asthma admissions for the February HAADs tended to be younger than the February average, the November HAADs showed an overrepresentation of adult patients (for both sexes) relative to the distribution for November.

We applied a binomial test of proportions to assess whether, within a given age group, the fraction of male patients was significantly different from 0.5. Differences (significant at the 0.05 level) were found in virtually all age categories, with the exception of when one restricts the analysis to HAADs only (the lack of statistical significance in such cases is likely due to the smaller number of cases).

### Prediction of asthma hospital admissions

As mentioned previously, eight different regression models were fitted, which differed in terms of which groups of predictor variables to use, and which period to run (see S2 and S3 Tables). In order to remain robust to the more sensitive elements, we summarise conclusions over a range of models (S4 to S11 Tables and S6 to S13 Figs). The regression was performed for the age-group 0-64, since the 65+ age group shows little response to the November (seasonal allergy) peak. The most “informative” models (having the greatest predictive skill in out-of-sample data) were model 3 & 7 (S2 and S3 Tables). These models included the cyclical day-of-year effect, non-linear effects related to meteorological, pollen and air quality variables (air quality variables appear in Model 7 but not in Model 3), the impact of thunderstorms and day-of-week effects; these models did not include interaction terms between the thunderstorm and pollen variables. The inclusion of such interaction terms added substantial complexity but did not improve the out-of-sample predictive skill. Model 3 can be expressed as:

$$\begin{array}{ccc} y_{i} & = & c+S\left({\text{DOY}}_{i}\right)+s\left({\text{RH}}_{\text{rl},i}\right)+s\left({\text{RH}}_{\text{dv},i}\right)+s\left({\text{TM}}_{\text{rl},i}\right)+s\left({\text{TM}}_{\text{dv},i}\right)+s\left({\text{PR}}_{i}\right)\\ & + & s\left({\text{NS}}_{i}\right)+s\left({\text{EW}}_{i}\right)+s\left({\text{GR}}_{i}\right)+s\left({\text{NG}}_{i}\right)+s\left({\text{GR}}_{\text{lg},i}\right)+s\left({\text{NG}}_{\text{lg},i}\right)\\ & + & \beta_{\text{Mo}}\cdot I_{\text{Mo},i}+\beta_{\text{Tu}}\cdot I_{\text{Tu},i}+\beta_{\text{We}}\cdot I_{\text{We},i}+\beta_{\text{Th}}\cdot I_{\text{Th},i}+\beta_{\text{Fr}}\cdot I_{\text{Fr},i}+\beta_{\text{Sa}}\cdot I_{\text{Sa},i}\\ & + & \beta_{\text{TS}}\cdot I_{\text{TS},i}+e_{i}, \end{array}$$

where *y*_*i*_ is the asthma admissions rate (per 100,000 population) on day *i*, *c* is a constant offset parameter, *s*(*x*) is a mean-zero cubic spline (for predictor variable *x*), *S*(*x*) is a mean-zero cyclical cubic spline, DOY_*i*_ is the day of the year, RH is the relative humidity, TM is the temperature, PR is the precipitation, NS is the north-south wind component, EW is the east-west wind component, GR is the grass pollen concentration, NG is the non-grass pollen, *β*_*Y*_ is a parameter associated with variable *Y*, *I*_*X*_ is an indicator variable (equal to 1 when condition *X* is true, and 0 when it is false) and *e*_*i*_ is the residual (assumed to be independent, following a Gaussian distribution with mean 0 and constant variance). The subscript *Y*_rl,*i*_ denotes a backward-looking rolling 14-day mean for variable *Y*, *Y*_dv,*i*_ denotes the daily deviation from this rolling mean, and *Y*_lg,*i*_ denotes a lagged mean of the previous three days before day *i*. The conditions for binary variables are encoded as follows and appear as subscripts: thunderstorm (TS), Monday (Mo), Tuesday (Tu), Wednesday (We), Thursday (Th), Friday (Fr) and Saturday (Sa). For the categorical variables of weekday and the thunderstorm indicator, the reference levels were Sunday and “no thunderstorm”. Model 7 also has terms representing smoothed effects of particulate matter and ozone. We note that the model-fitting process had the ability to implicitly exclude uninformative variables, and the east-west wind component (EW) was found to contribute little predictive information when all other variables were considered.

When looking across the whole year (Models 1, 5) the seasonal cycle in admissions (Fig 1A) was the most important explanatory variable for observed asthma admission rates, followed by the rolling mean temperature and the rolling mean humidity. The peaks in the annual seasonal cycle reflect those shown in Fig 1 (which was estimated without other covariates); for the October-December period (Models 2-4, 6-8), admissions peaked between October 27^th^ and November 6^th^. The effect of the fortnightly rolling-mean temperature shows increased asthma admissions at around 20 degrees, and decreased admission rates below 15 degrees or above 25 degrees. For the rolling-mean relative humidity, admission rates were higher above 55-60% RH, and lower below this point.

For the models fitted with data from the October-December period only (models 2-4 & 6-8), rainfall explains a relatively larger part of the variation (c.f. Models 1, 5), with admissions exacerbated for days with 15-25 mm rainfall in particular. Thunderstorms were associated with an increase in asthma admission rates, estimated at in around 1-2 additional admissions (in a population of 4 million) on thunderstorm days. When the interaction between thunderstorms and the pollen variables are allowed, thunderstorms without pollen led to a decrease (of roughly 1.4 fewer admissions) in admission rates, whereas high levels of grass pollen over the three days preceding the thunderstorm were associated with increased admissions (roughly 3-5 additional admissions).

Considering the October-December period, the effect of the daily grass and non-grass pollen was not consistent across the eight models. The lagged pollen variables (averaged over 3 days prior to the given date), however, were associated with increased asthma admission rates. When average grass pollen concentration of the previous three days exceeded 70 grains/m^3^, the exacerbation became significant, plateauing at around 4 additional admissions per day for mean concentrations of 100 grains/m^3^ or more. Increased asthma admissions were associated with periods when the mean non-grass pollen concentrations over the previous three days ranged between 500 and 1300 grains/m^3^.

None of the air quality variables showed a consistent association with increased asthma admissions, despite the well-characterised impacts of fine particulate matter and ozone in particular [38, 39]; this is likely due to limitations of the monitoring network in estimating population-level exposure. This result was found both when using comparing admissions with measurements from one stable site (Alphington) and when averaging over data from available sites (not shown).

The day-of-week effect was apparent in all models, with Sunday and Monday having the highest admissions (the effect size was around 2-3 more admissions on Sunday and Monday than on Thursday or Friday). The discrepancy between the different weekdays varied throughout the year, with the discrepancy between Sunday/Monday and the rest of the week being greatest during the months of February and March (S16 Fig), and smallest in January; we note that January is the main summer holiday period for Melbourne residents.

In the GAMs (S6 to S13 Figs) there was evidence of non-linear relationships between some of the explanatory variables and the observed admission rates. Apart from the annual seasonal cycle (Fig 1A), considerable non-linearity was exhibited for precipitation, the rolling mean temperature and the rolling mean humidity.

**Fig 3:**
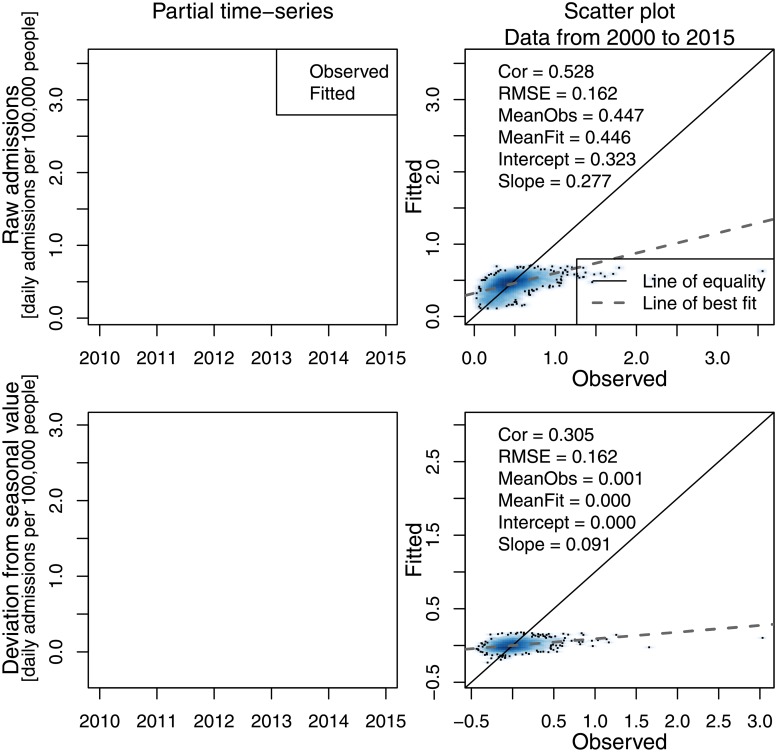
Modelled and observed population-normalised asthma admission rates (above), and deseasonalized equivalents (below). Upper row: Time-series of observed population-normalised asthma-related hospital admissions (black) and the corresponding predicted values from the model (red). Lower row: the same time-series minus the seasonal mean. Left column: a time-series of these data is shown for a shorter period (2010-2015) to highlight the seasonality. Right column: a scatter plot of modelled versus observed values for the full data series (2000-2015), shown as a two-dimensional density plot (with outliers given as points); these panels also show some summary statistics and the least-squares linear model fit.

Considering the output of one of the fitted models (model 3, S3 Table), we see that the fitted values are dominated by the seasonal cycle (Fig 3). The fitted model accounts for 53% of the variation in the observations, although once the seasonal cycle is subtracted from both the observed and fitted values, the correlation fell to 31%. Furthermore, the model had little skill in predicting the extreme cases.

A high asthma episode of particular interest is the 21^st^ November 2016, as it was the most extreme on record. While our admissions dataset does not extend into 2016, we have applied each of the eight models to the environmental covariates of that day. In the case of two of the models (models 1 and 5, which were trained on the full annual time-series and did not rely on pollen), the predicted admission rate for this day was in the top 1% of predicted admission rates, while for the other models it was on par with the top 3%-6% of predicted admission rates. However, the models showed no skill in predicting the magnitude of the event (which was unprecedented in scale); all predicted between 24.6 and 28.5 admissions. This pales in comparison to the 476 excess admissions (above the seasonal average) as reported by the Victorian Inspector General for Emergency Management [40]; n.b. the figure of 476 excess admissions is for a slightly larger area, encompassing both the Melbourne and Geelong regions (the Greater Melbourne Region had a population of 4.485 million in 2016, and Geelong population of 0.279 million at a national census in August 2016 [41]) and a longer period (30 hours vs. 24 hours). Regardless, all eight models dramatically underestimated the number of admissions.

### High-asthma episodes

Table 1 lists the 16 most extreme HAADs during the 25-year period. Of these, 10 were during the October-December period, one fell in mid-September and the remaining five occurred during the last two weeks of February. All six HAADs in February occurred on a Sunday (two dates) or a Monday (four dates). Two of the HAADs (2003-11-20, 2010-11-25) have previously been described as “thunderstorm asthma” events [23, 24].

Thunderstorms in Melbourne are also highly seasonal, with an average of roughly 1.5-2 thunderstorm days per month between November and January. This compares to 0.5 thunderstorm days per month or fewer from April to September [42] and around 1.3 thunderstorm days per month in February, March and October. However, days with thunderstorms were still over-represented among the HAADs (44% vs. 12% for the full dataset, *p* < 0.001 for the χ^2^-test for equality of proportions), as were days with at least 2 mm of rainfall (56% vs. 19%, *p* < 0.001) and winds over 15 km/h from the north (56% vs. 26%, *p* = 0.05). Considering the HAADs occurring in the October-December period, pollen concentrations above 50 grains/m^3^ were over-represented in the daily concentration (50% vs. 24% for grass, *p* = 0.11), the average over the previous three days (90% vs. 26% for grass, *p* < 0.001), and the previous seven days (70% vs. 27% for grass, *p* < 0.01); none of the differences for non-grass pollen were statistically significant.

### Prediction of high-asthma episodes

In light of the poor skill of the GAMs in predicting extreme asthma admissions cases (Fig 3), we now consider the skill of the logistic regression model or a gradient boosting model in the prediction of HAADs. For each of the 100 random splits between the testing and training datasets, the number of predicted and observed non-HAADs and HAADs was calculated. The frequency of HAADs in the training set was used to set the threshold response values of each model to discretise the modelled data (i.e. if there were four HAADs present in the test set, the dates with the four highest predicted probabilities were taken to be the “modelled HAADs”). Averaging across 100 replicates, we see that the models rarely correctly identified HAADs, and most of the predicted HAADs were not actually HAADs (Table 2). Gradient boosting appeared the more skilful of the two approaches, although both methods had low true positive rates (13% for the logistic regression and 17% for the boosting).

**Table 2:** Mean number of predicted and observed HAADs from the October-December period, averaging over randomly selected test datasets. The left half of the table shows values for the logistic regression, while the right half of the table shows values from gradient boosting. Note that the “observed” number of HAAD/non-HAAD days has a fractional component, due to the averaging over randomly partitioned testing/training sets.

		Predicted			
		Logistic regression		Boosting	
		Not HAAD	HAAD	Not HAAD	HAAD
Observed	Not HAAD	401.51	2.33	402.16	2.22
	HAAD	2.48	0.38	2.36	0.50

### Discussion

Although multivariate statistical regression was successful in simulating seasonal variation in asthma admissions, it explained only a small fraction of the variation around the seasonal average. These findings echo those of Soyiri et al. [43], who used similar regression techniques to examine two years of asthma admissions data in London. The statistical classification of HAADs vs. non-HAADs showed similarly poor skill, which is partly a result of the rare nature of HAADs. The small number of cases makes it difficult to build up a robust representation of conditions conducive for such an effect. It is possible that other predictive techniques (e.g. based on prognostic weather models, coupled to an atmospheric-transport model for pollen) may prove more successful. The negative results notwithstanding, this study presents a number of other useful findings regarding the most predictable triggers of asthma at a community level.

One element that was not considered in this study was exposure estimation, and this this may be a fruitful direction of study in future. Indeed, the November 2016 event was likely to have been made more acute as a result of the storm front passing over the city at 17:30h on a weekday [40].

The strength of the associations may have been weakened by a degree of temporal mismatch between some of the key variables in the regression analysis. Some variables were midday snapshots (most of the meteorological parameters), others were accumulated from midnight to midnight (admissions, thunderstorms, rainfall), and the pollen data were averaged from 16:00h one day to 16:00h the next day. The limited representativity of midday snapshots can be seen in the temporal profiles of the meteorological variables over the HAAD days (S17 to S19 Figs); however a sensitivity analysis (not shown) using daily means rather than midday snapshots yielded virtually the same conclusions. We also note that there can be a degree of delay between the onset of asthma and asthma admissions. On the 25^th^ of November 2010, the most extreme HAAD in the time-series presented here, a storm front reached Melbourne around 20:00h the previous evening (24/11/2010), yet most of the admissions were recorded the following day [24, 25].

Seasonality in health care attendances for asthma is well recognised, although the size and timing of peaks varies by age-group and geographic location. Spring peaks are usually attributed to aeroallergens, winter peaks to increases in respiratory viral infections in the community, and autumn peaks have been associated with the return to school period in children [2, 5, 44]. Consistent with the wider literature, peaks in the study occurred in all seasons with the lowest asthma admission rate in the summer months of December and January. Asthma is a heterogeneous syndrome with many different causal factors, precipitants and clinical phenotypes. For example, asthma that begins in childhood is commonly allergic in origin, while asthma that initiates in adulthood is less commonly associated with an allergic predisposition or immediate allergic responses to environmental triggers [45, 46]. The differences in the seasonal patterns of presentations in different age-groups are likely to reflect the different clinical phenotypes of asthma. There was some evidence for differences in the patient cohorts for HAADs relative to all days; the admitted patients tended to be younger during February HAADs than the Feburary norm, while the opposite was true for HAADs in November. While asthma exacerbations during thunderstorm epidemics is strongly associated with an acute allergic response [47], it is not possible to identify the underlying reason for asthma symptoms in any individual case from hospital administrative datasets. Training the models on the subset of asthma admissions restricted to those caused by acute allergic responses would likely have improved the capacity of the statistical model to predict high asthma admission days associated with thunderstorms. This, however, was not possible due to the small number of cases with this particular diagnosis, and the considerable temporal variation with which this diagnosis was applied (much more in some years than others).

After the seasonal cycle the most important predictor of asthma admission rates was the moving-average relative humidity; asthma rates were higher after humid periods. One possible explanation is that this is a statistical artefact, arising because trends in the moving-average relative humidity are a marker for year-to-year variation in the timing of the peaks in asthma admissions. Another interpretation is that this is a meaningful association. It may reflect a change in behaviour (more time being spent indoors, thus increased exposure to indoor aeroallergens such as dust mites), or a change in either indoor or outdoor aeroallergens, such as dust mites or fungal spores. Fungal sensitisation and asthma severity show a close association [48]. Spores for some fungal taxa are found more often in humid conditions [49], and spores of some outdoor fungal taxa have been associated with increased asthma admissions in children and adolescents in Melbourne [50].

The effect of behaviour may be reflected in the differential admission rates for the days of the working week throughout the year. In Melbourne, the month of January generally coincides with summer holidays (for all school children and many adults). Also potentially reflecting behaviour patterns, all the HAADs from the return-to-school period fell on either a Sunday or a Monday (Table 1). There appeared to be a non-linear response for rainfall, leading to increased admission as rainfall increased until about 20mm/day and then appearing to fall thereafter (n.b. the frequency of such intense rainfall declines substantially at this point); the declining asthma rates on very wet days may be either due to causative reasons (e.g. wet deposition of outdoor aeroallergens) or behavioural changes affecting time spent outside.

The impact of pollen was complicated; associations were found between asthma admission rates and not only the daily grass pollen concentration, but also the average concentration from the previous three days. This could reflect sensitisation due to recent exposure to the aeroallergen [51], or it might reflect other delayed allergic or non-allergic pathophysiological pathways. While high levels of non-grass pollen in the prior three days were associated with a slight elevation in asthma admission rates, the total daily non-grass pollen had no clear impact on asthma admissions; this taxonomic grouping is most likely too broad to provide much information.

Thunderstorms by themselves were seen to have a mild impact on asthma admission rates (associated with an increase of around 10%). The evidence for the modulating effect of pollen was somewhat ambiguous, with a stronger association to the lagged pollen levels than the daily pollen concentration. Clearly the impact of storms on asthma varies, and the treatment in this study is extremely simple (a day either did or did not have a thunderstorm). A more nuanced classification of storm types may improve our understanding of their impacts on respiratory health.

The air pollutants examined (O_3_ and PM_2.5_) are well recognised precipitants of asthma exacerbations and have been documented to be associated with admissions for asthma in multiple studies the world over [38, 39]. However in this analysis they were not associated with asthma admissions, either for concentrations from a single site or a geographical average. The relatively limited air monitoring network in Melbourne could have resulted in exposure misclassification in individual patients, which would have biased results towards the null.

Thunderstorm asthma is generally considered a rare event that makes only a small contribution to the total burden of disease from asthma [11]. However at least six thunderstorm asthma events have been reported in Melbourne over the last 30 years, including the extreme event of the 21^st^ November, 2016 [13, 21–25]; this contradicts the view of its rarity and that it may instead be a semi-regular seasonal occurrence, with an average incidence of once every five years in this region. In general (ignoring the influence of thunderstorms), the rate of asthma epidemics depends on the threshold used to demarcate such incidents; considering those cases listed in Table 1, we find an average frequency of two events per three years.

Despite the lack of skill in predicting thunderstorm asthma episodes, one can still characterise some of their features, at least for Melbourne. All such incidents have occurred in the second to the fourth weeks of November and synoptic-scale changes in wind, temperature and humidity [25]. Seasonal grass pollen in Melbourne has been associated with seasonal spring rainfall [52], and grass phenology may be broadly diagnosed from remote-sensing data [53]. Such elements may form part of an operational warning system. However at this stage the science has not yet advanced to the stage where the interactions between clinical characteristics with season, palynology, meteorology and individual clinical factors are understood sufficiently to enable a valid and reliable daily forecasting system during these time periods. It is clear that February and November are especially high risk months for high asthma admission days in Melbourne and other parts of Australia (S15 Fig), and that those that occur in late spring are frequently associated with thunderstorms. Public health messaging prior to and during these high risk time periods could help individuals at risk, their treating doctors and health services to be better prepared to manage an event, especially those who are aware that they have allergies to pollen or other aero-allergens.

## Appendix 1

### The choice of thunderstorm indicators

Unfortunately there is no unequivocal method for identifying thunderstorms. The Americal Meteorological Society’s Glossary of Meteorology offers the following definition [55]:

“In general, a local storm, invariably produced by a cumulonimbus cloud and always accompanied by lightning and thunder, usually with strong gusts of wind, heavy rain, and sometimes with hail.

It is usually of short duration, seldom over two hours for any one storm. A thunderstorm is a consequence of atmospheric instability and constitutes, loosely, an overturning of air layers in order to achieve a more stable density stratification. A strong convective updraft is a distinguishing feature of this storm in its early phases. A strong downdraft in a column of precipitation marks its dissipating stages …A unique quality of thunderstorms is their striking electrical activity.”

To add to the complexity of defining thunderstorms, there is some evidence to suggest that only certain types of thunderstorms risk exacerbating asthma at a population-level [12]. Storms leading to outflow gusts, and with winds coming from the source regions for putative bioaerosol triggers have elements that may lead to thunderstorm asthma, however the mechanisms are as yet unclear (see, for example, [56]).

In this study, we have not made any assumptions about what types of thunderstorms may be more likely to instigate asthma exacerbations. The long time-series of 25 years of admissions, meteorological and pollen data allows us insights that might not be possible with a shorter data-set, however it brings with additional challenges.

First, the time-series is too long to make manual identification thunderstorms possible, such as was done in Marks et al. [12]. Second, different data-sets that may allow us to identify thunderstorms (henceforth “thunderstorm indicators”) exist for different periods, with limited overlap. Third, there is no “gold standard” reference in this case. As such, we aim to select the best thunderstorm indicator available, for the longest possible period.

#### GPATS lightning strike count around Melbourne

The Global Positioning and Tracking Systems (GPATS) Pty. Ltd. is a commercial company runs a network of lightning tracking equipment. A time-series of lightning strike counts within 50 km of the Melbourne GPO was purchased. The data are given as the total number of strikes per day, and runs from 2003 to 2010. For the purposes of this part of the study, a thunderstorm was deemed to have occurred if more than 2 lightning strikes were reported on a given day.

#### Gridded GPATS lightning strike counts

A gridded dataset of GPATS lightning stike counts [57] was accessed through the Austrlian National Computation Infrastructure (NCI). The dataset covers the period 2008 to 2015 on a 0.75° × 0.75° spatial resolution (roughly 75 km × 75 km) and at hourly temporal resolution. To improve match with the GPATS lightning strick data around Melbourne (above), we restricted the data the 2 × 2 grid-cells closest to Melbourne GPO (or equivalently, those grid-cells for which the centre lies within 75 km of this location).

#### Melbourne airport METAR/SPECI

Observer reports used for aviation reporting (METAR/SPECI) from Melbourne Airport were obtained from the Australian Bureau of Meteorology for the period 1990-2015, although there was a change in the way the data were archived around 1999. The METAR/SPECI reports are provided in a very specific encoding. For this indicator, thunderstorms were deemed to have occurred if the observer reported this (coded as TS).

#### Thunder heard at Melbourne airport

The observer reports from Melbourne Airport also included whether any thunder was heard. For this indicator, thunderstorms were deemed to have occurred on days when a “thunder heard” report was noted by the observer.

#### High CAPE at Melbourne airport

Weather balloons record wind speed/direction, temperature, pressure and humidity throughout the vertical profile. Such profiles allow for the calculation of CAPE (convective available potential energy), a widely used measure for the potential for thunderstorm formation. Thunderstorms require two key ingredients to form: a large amount of moist air, and a lifting mechanism. The lifting mechanism can be orographic (air passing over mountains) or convective. The CAPE is a measure of the convective potential energy. Values over 5 J/kg are considered “high”.

Weather balloons are released at Melbourne airport twice daily, at 00 H UTC (10 am Australian Eastern Time) and at 12 H UTC (10 pm Australian Eastern Time). These are archived (together with weather soundings worldwide) by the University of Wyoming, and soundings for the period August 1999 to December 2011 were downloaded from from their website [58]. These files also include a number of summary statistics, and among them was the CAPE. For this indicator, a thunderstorm was recorded when the CAPE was above 5 J/kg.

#### Strong rain rates

One common feature of thunderstorms is intense rain rates. Half-hourly observations from all Australian automatic weather stations were purchased from the Bureau of Meteorology. We calculated hourly rain-rates for the 16 stations within 75 km of the Melbourne GPO and which had been operational since 2000. For this indicator, thunderstorms were deemed to have occurred on days when at least 25% of these stations reported rain rates of 3 mm/hour.

#### The Australian Bureau of meteorology’s severe storm archive

The Australian Bureau of Meteorology’s maintains a database of severe weather events [59]. One cannot select “thunderstorms” specifically (available options are: Rain, Hail, Wind, Tornado, Lightning, Waterspout, Dust devil). We extracted reports of all storm types for Victoria for the period 1990-2015. These were then filtered (based on the geographical coordinates of the report location) to a radius of 75 km around the Melbourne GPO. For this indicator, thunderstorms were deemed to have occured on a given day if at least one report appeared within this region.

#### Comparison of thunderstorm indicators

It was possible to calculate all of the thunderstorm indicators for a total of 1084 days, spanning three years (2008-2010, with data from 12 days missing). During this overlap period, we calculated the agreement, as measured by the “Equitable Threat Score”, henceforth the ETS [60]. The ETS is a measure of the skill of a binary (true/false) prediction, adjusting for the fact that some of the apparent skill may be due to chance. The ETS has been shown to be subject to bias, and the ETS tends to increase with the total number of true “events” [60]. As such we acknowledge the limitation; it is used here to provide some guidance about which thunderstorm indicator to use, rather than an absolute metric of skill (especially since there is no “reference” set). An ETS value of 1.0 indicates perfect agreement, and the statistic ranges from −1/3 to 1.0.

The strongest agreement can be seen between the GPATS around Melbourne, the gridded GPATS and the METAR (see S1 Table). It can be argued that lightning is a necessary condition of thunderstorms (which measured by the GPATS data), and the METAR provides us the closest proxy for these data. The METAR indicator is preferable since it provides us with the longest possible time-series, of which we choose to use the 1999-2015 period. This is because the change in archiving method for these METAR data during 1999 led to some inconsistencies in the data, such that we had less confidence in the 1990-1998 period.

## Appendix 2

### Calculation of effect sizes

S4 to S11 Tables, respectively, results of regression models 1 to 8 (see S3 Table for further details of each model). They show the *t*-value (the estimate of the coefficient, normalized by the associated standard error), the associated *p*-value from the hypothesis test that this term is non-zero, and an estimate of the “effect size”.

The motivation for presenting an estimate of the effect size is that there is distinction between statistical significance and practical significance. If one has an enormous dataset, one can typically find patterns of high statistical significance but with only trivial practical impact. By contrast, in a very small dataset, even effects of large practical significance may not be demonstrable statistically (using the Frequentist statistical approach). Also relevant is that the presentation of raw regression coefficients is, by itself, uninformative, since variables are generally on different scales and may have different units.

The effect sizes presented in S4 to S11 Tables represent an estimate of the number of daily asthma admissions associated with particular causes, assuming a population of 4.0 million (n.b. Melbourne’s population surpassed 4.5 million in 2015; [29]). For numerical predictor variables (see S2 Table), this was done by calculating the 0.95 quantile of each predictor variable, multiplying this by the regression coefficient obtained, and then multiplying again by 40 (the number of cases in a population of 4.0 million will be 40 times the rate per 100,000 individuals); the choice of the 0.95 quantile means that this will be on the upper end of the effect-size distribution, however it provides a yardstick with which to compare. For binary or categorical variables, the effect size was simply the regression coefficient multiplied by 40.

## Supporting information

S1 Fig Maps of the region. Maps of the region. Panel A: Australia, with the state Victoria highlighted. Panel B: Victoria, with the state capital, Melbourne, labelled. Panel C: The greater Melbourne metropolitan region, with the location of Melbourne Airport illustrated with an ascending aeroplane symbol. Panel D: The location of the two pollen sampling sites relative to the centre of the city (the Melbourne General Post Office). Panels A, B and D were generated using Google Maps (locations of the samplers on panel D were added manually). Panel C derives from pp. 5 of [54], with the aeroplane symbol added manually.
(PDF)

S2 Fig Locations of air quality monitoring sites. Sites are shown relative to the Melbourne Central Business Distict. The Alphington site is indicated. See S1 Fig for the broader geographical context.
(PDF)

S3 Fig Raw (unadjusted) number of admissions per diagnostic category. The top-left and top-right panels show daily admission numbers for admission categories J45 and J46, respectively, following the World Health Organisation’s International Classification of Diseases (ICD) 10^th^ revision (ICD-10). The bottom panel displays the sum of the daily counts for these two diagnostic codes. The J45 category refers to asthma and includes predominantly allergic asthma, nonallergic asthma and mixed or unspecified asthma. The J45 classification excludes acute severe asthma, chronic asthmatic (obstructive) bronchitis or chronic obstructive asthma, eosinophilic asthma, lung diseases due to external agents and status asthmaticus. The J46 category refers to status asthmaticus, includeing acute severe asthma.
(PDF)

S4 Fig Population normalised daily admissions for the 25 year period considered. (PDF)

S5 Fig Residuals in the admissions time-series. Residuals in the admissions time-series. **A**: residuals normalised by the 31-day centred running mean and standard deviation. **B**: residuals normalised by the annual mean and standard deviation. **C**: population-normalised residuals minus the seasonal component (Fig 1 in the main text).
(PDF)

S6 Fig Smoothing spline fits for the non-linear terms for model 1. Smoothing spline fits for the non-linear terms for Model 1. The solid black line shows the estimated fit; this is surrounded by a grey area, which represents 2 standard errors above and below the estimate. These confidence bands include the uncertainty about the overall mean (thus each has an average of 0.0). The blue dashed horizontal line was drawn at *y* = 0.0 on each plot. The small vertical dashes from the bottom of each panel show the distribution of values of that variable. Note that the scale on the *y*-axis differs between the panels. To estimate the corresponding “effect size” (i.e. the additional number of admissions associated with each term in a population of 4 million), one multiplies the functions by 40. See also S4 Table.
(PDF)

S7 Fig Smoothing spline fits for the non-linear terms for model 2. See also S5 Table and the captions of S4 Table and S6 Fig for further details.
(PDF)

S8 Fig Smoothing spline fits for the non-linear terms for model 3. See also S6 Table and the captions of S4 Table and S6 Fig for further details.
(PDF)

S9 Fig Smoothing spline fits for the non-linear terms for model 4. See also S7 Table and the captions of S4 Table and S6 Fig for further details.
(PDF)

S10 Fig Smoothing spline fits for the non-linear terms for model 5. See also S8 Table and the captions of S4 Table and S6 Fig for further details.
(PDF)

S11 Fig Smoothing spline fits for the non-linear terms for model 6. See also S9 Table and the captions of S4 Table and S6 Fig for further details.
(PDF)

S12 Fig Smoothing spline fits for the non-linear terms for model 7. See also S10 Table and the captions of S4 Table and S6 Fig for further details.
(PDF)

S13 Fig Smoothing spline fits for the non-linear terms for model 8. See also S11 Table and the captions of S4 Table and S6 Fig for further details.
(PDF)

S14 Fig Quantile-quantile plot of the normalised residual admission rates. Quantile-quantile plot for the residuals normalised by the 31-day centred running mean and standard deviation (see Fig A). The horizontal line, at a value of *z* = 4.5, shows the threshold above which dates were labeled as “high asthma admissions days” (HAADs). Above this threshold, points were drawn in grey if they fall in the October to December period and in black at any other month of the year.
(PDF)

S15 Fig Monthly numbers of HAADs and average pollen concentration in Melbourne. The monthly distribution of HAADs and mean monthly pollen concentration as measured in Melbourne. Grass pollen data shown here are based on Erbas et al. [61].
(PDF)

S16 Fig Day-of-week effects on asthma admission rates by month of year. The average admissions per day-of-week, for each month of the year, across the full 1990-2015 period.
(PDF)

S17 Fig Meteorological data from selected HAADs (part 1). Half-hourly meteorological data recorded at Melbourne Airport (by the Bureau of Meteorology) on four high asthma admission dates in the months of October through to December (see Table 1 in the main text). The cumulative precipitation is reset at 9 am each day. Wind direction is shown when data were available at the 30 minutes past the hour. Continued in S18 and S19 Figs.
(PDF)

S18 Fig Meteorological data from selected HAADs (part 2). See S17 Fig for further details.
(PDF)

S19 Fig Meteorological data from selected HAADs (part 3). See S17 Fig for further details.
(PDF)

S1 Table Thunderstorm metrics compared with the Equitable Threat Score. The seven thunderstorm metrics considered, compared using the Equitable Threat Score (ETS), a measure of the correspondence between the classification of thunderstorm events, corrected for chance agreement. A value of 1.0 indicates perfect agreement, and the statistic ranges from −1/3 to 1.0. The column on the right-hand side show the total number of events indicated during the three-year period.
(PDF)

S2 Table Details of variables included in the regression analysis. For each variable, details are provided of their respective spatial and temporal resolutions. Abbreviations: BoM = Australian Bureau of Meteorology, MPC = Melbourne Pollen Count. Variable types are coded as follows: P = predictor, O = outcome, N = numerical, B = binary, C = categorical. The seasonal effect (the derived variable in the last row) is shown in Fig 1A in the main text.
(PDF)

S3 Table Summary of regression models considered. Summary of regression models considered. Abbreviations: Step = use step-wise variable selection?, YD = seasonal or day-of-year effect, RH = relative humidity, TM = temperature, PR = precipitation, NS = north-south wind component, EW = east-wst wind component, TS = thunderstorm, GR = grass pollen, NG = non-grass pollen, WK = weekday, * = decomposed into a rolling (backward-looking) 14-day mean and the daily deviation from this rolling mean, ^†^ = consider lagged variable over different times, A × B = interactions between variables A and B, Y = yes, N = no. When creating lagged variables for the pollen time-series, two variables were used in the regression (per pollen type—grass or non-grass): the value on the day of interest, and th means of the 3 days prior to the day of interest (i.e. not including the value of the day of interest).
(PDF)

S4 Table Summary of the fit for model 1. Summary of the fit for Model 1 (see S3 Table). The upper part of the table corresponds to the linear, binary or categorical terms whereas the lower half of the table provides information about fit of continuous variables that were allowed to vary non-linearly. The first column in both parts of the table gives the name of each term. Abbreviations used in the model summaries: yday = day-of-year effect; RH = relative humidity; TM = temperature; PR = precipitation; NS = north-south wind component; EW = east-wst wind component; TS = thunderstorm; GR = grass pollen; NG = non-grass pollen; WK = weekday; A:B = the interaction term between binary variable A and numerical variable B (set to zero when A is false); subscripts “rl” and “dv” = the rolling, backward-looking 14-day mean and the daily deviation from this rolling mean (respectively); terms WK_M_, WK_Tu_, WK_W_, WK_Th_, WK_F_, WK_S_ = the day-of-week effect (for Monday through to Saturday, respectively), relative to Sunday (positive values mean higher than the effect for Sunday); the EDF represents the estimated number of degrees of freedom for the non-linear terms. If the EDF is equal to 1.0, then the term shows no evidence of non-linearity; if the EDF is equal to 0.0, then the term has been dropped via shrinkage (implicit model selection). In the upper half of the table, the *t*-statistic and the associated *p*-value corresponds to a test for whether the parameter is non-zero. The “Effect size” column displays an estimate of the number of daily admissions, in a population of 4.0 million, associated with each term (see Appendix 2); the associated confidence interval accounts only for uncertainty in the regression coefficient, and does not address the range of the predictor variable. The lower part of the table shows information about the non-linear terms. The *F*-statistic and the associated *p*-value corresponds to a test for whether *all* the coefficients associated with this term are zero.
(PDF)

S5 Table Summary of the fit for model 2. Summary of the fit for Model 2 (see S3 Table). See the caption of S4 Table for further details.
(PDF)

S6 Table Summary of the fit for model 3. Summary of the fit for Model 3 (see S3 Table). See the caption of S4 Table for further details.
(PDF)

S7 Table Summary of the fit for model 4. Summary of the fit for Model 4 (see S3 Table). See the caption of S4 Table for further details.
(PDF)

S8 Table Summary of the fit for model 5. Summary of the fit for model 5 (see S3 Table). See the caption of S4 Table for further details.
(PDF)

S9 Table Summary of the fit for model 6. Summary of the fit for model 6 (see S3 Table). See the caption of S4 Table for further details.
(PDF)

S10 Table Summary of the fit for model 7. Summary of the fit for model 7 (see S3 Table). See the caption of S4 Table for further details.
(PDF)

S11 Table Summary of the fit for model 8. Summary of the fit for model 8 (see S3 Table). See the caption of S4 Table for further details.
(PDF)

## References

[1] Silverman R, Stevenson L, Hastings H. Age-related seasonal patterns of emergency department visits for acute asthma in an urban environment. Ann Emerg Med. 2003;42(4):577–586. doi: 10.1067/S0196-0644(03)00410-4 14520329 10.1067/s0196-0644(03)00410-4

[2] Chen C, Xirasagar S, Lin H. Seasonality in adult asthma admissions, air pollutant levels, and climate: a population-based study. J Asthma. 2006;43:287–292. doi: 10.1080/02770900600622935 16809242 10.1080/02770900600622935

[3] Zhang Y, Peng L, Kan H, Xu J, Chen R, Liu Y, et al Effects of meteorological factors on daily hospital admissions for asthma in adults: a time-series analysis. PloS ONE. 2014;9(7):e102475 doi: 10.1371/journal.pone.0102475 25019158 10.1371/journal.pone.0102475 PMC4097056

[4] Qiu H, Yu I, Tse L, Chan E, Wong T, Tian L. Greater temperature variation within a day associated with increased emergency hospital admissions for asthma. Sci Tot Environ. 2015;505:508–513. doi: 10.1016/j.scitotenv.2014.10.003 10.1016/j.scitotenv.2014.10.003 25461053

[5] Eggo R, Scott J, Galvani A, Meyers L. Respiratory virus transmission dynamics determine timing of asthma exacerbation peaks: Evidence from a population-level model. Proc Natl Acad Sci U S A. 2016;113:2194–2199. doi: 10.1073/pnas.1518677113 26858436 10.1073/pnas.1518677113 PMC4776522

[6] Kim K, Jahan S, Kabir E. A review on human health perspective of air pollution with respect to allergies and asthma. Environ Int. 2013;59:41–52. doi: 10.1016/j.envint.2013.05.007 23770580 10.1016/j.envint.2013.05.007

[7] Lincoln D, Morgan G, Sheppeard V, Jalaludin B, Corbett S, Beard J. Childhood asthma and return to school in Sydney, Australia. Public Health. 2006;120(9):854–862. doi: 10.1016/j.puhe.2006.05.015 16904142 10.1016/j.puhe.2006.05.015

[8] Cohen H, Blau H, Hoshen M, Batat E, Balicer R. Seasonality of asthma: a retrospective population study. Pediatrics. 2014;133:e923–e932. doi: 10.1542/peds.2013-2022 24616356 10.1542/peds.2013-2022

[9] Hoch H, Calatroni A, Szefler S. Validation Of Predictors For Fall Asthma Exacerbations In Inner City Children. In: B27. Viral Infection and Pediatric Asthma. Am Thoracic Soc; 2015 p. A2639–A2639.

[10] Teach S, Gergen P, Szefler S, Mitchell H, Calatroni A, Wildfire J, et al Seasonal risk factors for asthma exacerbations among inner-city children. J Allergy Clinl Immunol. 2015;135(6):1465–1473. doi: 10.1016/j.jaci.2014.12.1942 10.1016/j.jaci.2014.12.1942 PMC4461505 25794658

[11] Dabrera G, Murray V, Emberlin J, Ayers J, Collier C, Clewlow Y, et al Thunderstorm asthma: an overview of the evidence base and implications for public health advice. Q J Med. 2013;106:207–217. doi: 10.1093/qjmed/hcs234 10.1093/qjmed/hcs234 23275386

[12] Marks G, Colquhoun J, Girgis S, Hjelmroos Koski M, Treloar A, Hansen P, et al Thunderstorm outflows preceding epidemics of asthma during spring and summer. Thorax. 2001;56:468–471. doi: 10.1136/thorax.56.6.468 11359963 10.1136/thorax.56.6.468 PMC1746065

[13] Bellomo R, Giglotti P, Treloar A, Holmes P, Suphioglu C, Singh M, et al Two consecutive thunderstorm associated epidemics of asthma in the city of Melbourne. The possible role of grass pollen. Med J Aust. 1992;156:834–837. 1603007 10.5694/j.1326-5377.1992.tb136994.x

[14] Knox R. Grass pollen, thunderstorms and asthma. Clin Exp Allergy. 1993;1993:354–359. doi: 10.1111/j.1365-2222.1993.tb00339.x 10.1111/j.1365-2222.1993.tb00339.x 8334534

[15] D’Amato G, Liccardi G, D’amato M, Holgate S. Environmental risk factors and allergic bronchial asthma. Clin Exp Allergy. 2005;35(9):1113–1124. doi: 10.1111/j.1365-2222.2005.02328.x 16164436 10.1111/j.1365-2222.2005.02328.x

[16] Schaefer JT. Severe thunderstorm forecasting: A historical perspective. Weather and Forecasting. 1986;1(3):164–189. doi: 10.1175/1520-0434(1986)001%3C0164:STFAHP%3E2.0.CO;2

[17] Sofiev M, Bergmann KC, editors. Allergenic Pollen: A Review of the Production, Release, Distribution and Health Impacts. Springer; 2013.

[18] Haberle S, Bowman D, Newnham R, Johnston F, Beggs P, Buters J, et al The macroecology of airborne pollen in Australian and New Zealand urban areas. PLoS ONE. 2014;9(5):e97925 doi: 10.1371/journal.pone.0097925 24874807 10.1371/journal.pone.0097925 PMC4038531

[19] Graham B. Asthma Register, Charles Sturt University; 2016. Available from: http://science.csu.edu.au/asthma, accessed 2017-02-24.

[20] Victorian Department of Health and Human Services. Epidemic thunderstorm asthma forecasting; 2017. https://www2.health.vic.gov.au/public-health/environmental-health/climate-weather-and-public-health/thunderstorm-asthma/forecasting, last accessed 2017-12-21.

[21] Egan P. Weather or not. The Medical Journal of Australia. 1985;142:330–330. 3974498 10.5694/j.1326-5377.1985.tb113389.x

[22] McEvoy R. Thunderstorm associated epidemics of asthma. Med J Aust. 1992;157(5):352 1294131

[23] Erbas B, Akram M, Dharmage S, Tham R, Dennekamp M, Newbigin E, et al The role of seasonal grass pollen on childhood asthma emergency department presentations. Clin Exp Allergy. 2012;42:799–805. doi: 10.1111/j.1365-2222.2012.03995.x 22515396 10.1111/j.1365-2222.2012.03995.x

[24] Howden M, McDonald C, Sutherland M. Thunderstorm asthma—a timely reminder. Med J Aust. 2011;195:512–513. doi: 10.5694/mja11.11044 22060077 10.5694/mja11.11044

[25] Lindstrom SJ, Silver JD, Sutherland MF, Treloar A, Newbigin E, McDonald CF, et al Thunderstorm asthma outbreak of November 2016: a natural disaster requiring planning. The Medical Journal of Australia. 2017;207(6):235–237. doi: 10.5694/mja17.00285 28899321 10.5694/mja17.00285

[26] Ong EK, Singh MB, Knox RB. Seasonal distribution of pollen in the atmosphere of Melbourne: an airborne pollen calendar. Aerobiologia. 1995;11:51–55. doi: 10.1007/BF02136145

[27] Kappos AD, Bruckmann P, Eikmann T, Englert N, Heinrich U, Höppe P, et al Health effects of particles in ambient air. International journal of hygiene and environmental health. 2004;207(4):399–407. doi: 10.1078/1438-4639-00306 15471105 10.1078/1438-4639-00306

[28] ABS. Australian Historical Population Statistics, 2014; 2014. www.abs.gov.au/ausstats/abs@.nsf/Previousproducts/3218.0Main%20Features252014-15, accessed 24-04-2017.

[29] ABS. Estimated Resident Population—Greater Capital City Statistical Areas (GCCSAs); 2016. www.abs.gov.au/AUSSTATS/abs@.nsf/mf/3218.0, accessed 2017-02-03.

[30] Wood SN. Generalized Additive Models: An Introduction with R. Chapman and Hall/CRC; 2006.

[31] Wood SN. Fast stable restricted maximum likelihood and marginal likelihood estimation of semiparametric generalized linear models. J R Stat Soc Series B Stat Methodol. 2011;73:3–36. doi: 10.1111/j.1467-9868.2010.00749.x

[32] Natekin A, Knoll A. Gradient boosting machines, a tutorial. Front Neurorobot. 2013;7:21 doi: 10.3389/fnbot.2013.00021 24409142 10.3389/fnbot.2013.00021 PMC3885826

[33] R Core Team. R: A Language and Environment for Statistical Computing; 2015. Available from: https://www.R-project.org/.

[34] Hothorn T, Buehlmann P, Kneib T, Schmid M, Hofner B. mboost: Model-Based Boosting; 2016. R package version R package version 2.6-0. http://CRAN.R-project.org/package=mboost, accessed 2016-06-12.

[35] Lincoln D, Morgan G, Sheppeard V, Jalaludin B, Corbett S, Beard J. Childhood asthma and return to school in Sydney, Australia. Public health. 2006;120(9):854–862. doi: 10.1016/j.puhe.2006.05.015 16904142 10.1016/j.puhe.2006.05.015

[36] Johnston SL, Pattemore PK, Sanderson G, Smith S, Campbell MJ, Josephs LK, et al The relationship between upper respiratory infections and hospital admissions for asthma: a time-trend analysis. American journal of respiratory and critical care medicine. 1996;154:654–660. doi: 10.1164/ajrccm.154.3.8810601 8810601 10.1164/ajrccm.154.3.8810601

[37] Skobeloff E, Spivey W, Clair S, Schoffstall J. The influence of age and sex on asthma admissions. JAMA. 1992;268(24):3437–3440. 1460733

[38] Atkinson R, Kang S, Anderson H, Mills I, Walton H. Epidemiological time series studies of PM2. 5 and daily mortality and hospital admissions: a systematic review and meta-analysis. Thorax. 2014;7:660–665. doi: 10.1136/thoraxjnl-2013-204492 10.1136/thoraxjnl-2013-204492 PMC4078677 24706041

[39] Rice M, Guidotti T, Cromar K. Scientific evidence supports stronger limits on ozone. Am J Resp Crit Care Med. 2015;191:501–503. doi: 10.1164/rccm.201411-1976ED 25536009 10.1164/rccm.201411-1976ED

[40] IGEM. Review of response to the thunderstorm asthma event of 21-22 November 2016, Final Report. Inspector-General for Emergency Management, Victorian State Government; 2017.

[41] ABS. Community Profiles. Australian Bureau of Statistics; 2017.

[42] Kuleshov Y, De Hoedt G, Wright W, Brewster A. Thunderstorm distribution and frequency in Australia. Aust Meteorol Mag. 2002;51(3):145.

[43] Soyiri IN, Reidpath DD, Sarran C. Forecasting asthma-related hospital admissions in London using negative binomial models. Chron Respir Dis. 2013;10:85–94. doi: 10.1177/1479972313482847 23620439 10.1177/1479972313482847

[44] Wisniewski J, McLaughlin P AP andStenger, Patrie J, Brown M, El-Dahr J, Platts-Mills T, et al A comparison of seasonal trends in asthma exacerbations among children from geographic regions with different climates. Allergy Asthma Proc. 2016;37:475 doi: 10.2500/aap.2016.37.3994 27931303 10.2500/aap.2016.37.3994 PMC5080535

[45] Kim H, DeKruyff R, Umetsu D. The many paths to asthma: phenotype shaped by innate and adaptive immunity. Nat Immunol. 2010;11(7):577–584. doi: 10.1038/ni.1892 20562844 10.1038/ni.1892 PMC3114595

[46] Hekking P, Bel E. Developing and emerging clinical asthma phenotypes. J Allergy Clin Immunol Pract. 2014;2:671–680. doi: 10.1016/j.jaip.2014.09.007 25439356 10.1016/j.jaip.2014.09.007

[47] Nasser S, Pulimood T. Allergens and thunderstorm asthma. Curr Allergy Asthma Rep. 2009;9(5):384 doi: 10.1007/s11882-009-0056-8 19671382 10.1007/s11882-009-0056-8

[48] Denning D, O’Driscoll B, Hogaboam C, Bowyer P, Niven R. The link between fungi and severe asthma: a summary of the evidence. European Respiratory Journal. 2006;27:615–626. doi: 10.1183/09031936.06.00074705 16507864 10.1183/09031936.06.00074705

[49] Sabariego S, Diaz De la Guardia C, Alba F. The effect of meteorological factors on the daily variation of airborne fungal spores in Granada (southern Spain). Int J Biometeorol. 2000;44:1–5. doi: 10.1007/s004840050131 10879421 10.1007/s004840050131

[50] Tham R, Vicendese D, Dharmage SC, Hyndman RJ, Newbigin E, Lewis E, et al Associations between outdoor fungal spores and childhood and adolescent asthma hospitalizations. J Allergy Clin Immunol. 2016 10.1016/j.jaci.2016.06.046 27523960

[51] Boulet LP, Cartier A, Thomson NC, Roberts RS, Dolovich J, Hargreave FE. Asthma and increases in nonallergic bronchial responsiveness from seasonal pollen exposure. J Allergy Clin Immunol. 1983;71:399–406. doi: 10.1016/0091-6749(83)90069-6 6601124 10.1016/0091-6749(83)90069-6

[52] de Morton J, Bye J, Pezza A, Newbigin E. On the causes of variability in amounts of airborne grass pollen in Melbourne, Australia. Int J Biometeorol. 2011;55:613–622. doi: 10.1007/s00484-010-0361-x 20814699 10.1007/s00484-010-0361-x

[53] Reed B, Brown J, VanderZee D, Loveland T, Merchant J, Ohlen D. Measuring phenological variability from satellite imagery. J Veg Sci. 1994;5(5):703–714. doi: 10.2307/3235884

[54] ABS. Greater Capital City Statistical Areas. A Statistical Geography Fact Sheet.; 2013. www.abs.gov.au/websitedbs/d3310114.nsf/home/asgs+fact+sheets, accessed 2017-01-26.

[55] AMS. Thunderstorm. Part of “Glossary of Meteorology, 2nd Edition”; 2012. http://glossary.ametsoc.org/wiki/Thunderstorm, accessed 2017-01-26.

[56] Grundstein A, Sarnat SE. Meteorological Mechanisms Explaining Thunderstorm-Related Asthma. Geography Compass. 2009;3:45–63. doi: 10.1111/j.1749-8198.2008.00195.x

[57] Bunn R, Ramsay H. Lightning Stroke Counts on ECMWF Era Interim Grid; 2016. http://climate-cms.unsw.wikispaces.net/Lightning+Stroke+Counts+on+ECMWF+Era+Interim+Grid, accessed 2017-01-26.

[58] University of Wyoming. Atmospheric soundings; 2016. http://weather.uwyo.edu/, accessed 2017-01-26.

[59] of Meteorology AB. Severe Storms Archive; 2017. www.bom.gov.au/australia/stormarchive, accessed 2017-01-26.

[60] Hamill TM, Juras J. Measuring forecast skill: is it real skill or is it the varying climatology? Q J R Meteorol Soc. 2006;132:2905–2924.

[61] Erbas B, Abramson M, Dharmage S, Hyndman R, Taylor P, Bardin P, et al. Weekly pollen count data collected for the Melbourne Air Pollen Children and Adolescent Health (MAPCAH) study; 2014. http://dx.doi.org/10.4227/05/5344E4980DAF6, accessed 10 January 2017. Published on the Australian Centre for Ecological Analysis and Synthesis (ACEAS) data portal, part of the Terrestrial Ecosystem Research Network (TERN).

