# Supplementary figures and images for: Seasonal asthma in Melbourne, Australia, and some observations on the occurrence of thunderstorm asthma and its predictability
Source: PLoS One. 2018 Apr 12;13(4):e0194929. doi: 10.1371/journal.pone.0194929 (PMC5896915; doi:10.1371/journal.pone.0194929)

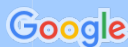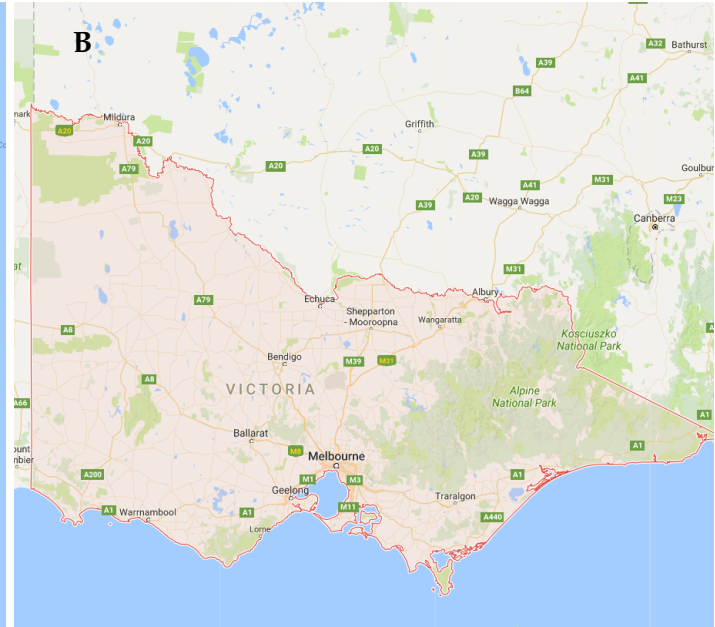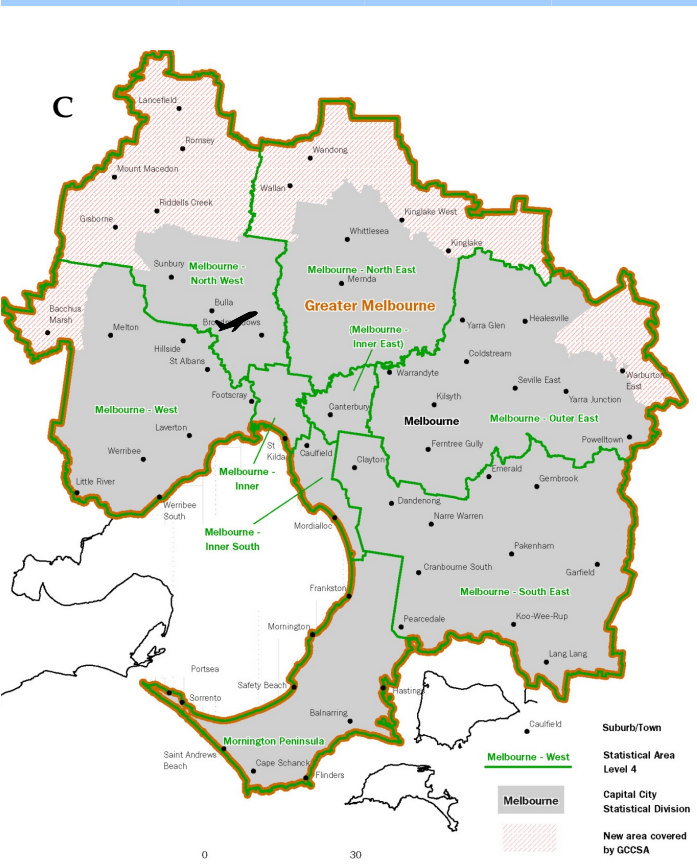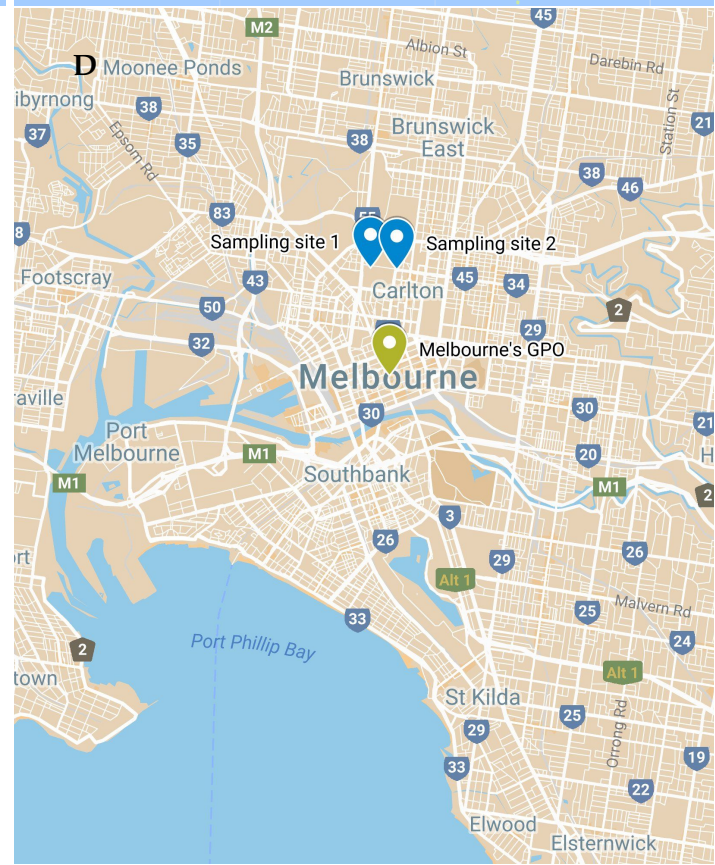

Supplement: S1 Fig — Maps of the region. Panel A: Australia, with the state Victoria highlighted. Panel B: Victoria, with the state capital, Melbourne, labelled. Panel C: The greater Melbourne metropolitan region, with the location of Melbourne Airport illustrated with an ascending aeroplane symbol. Panel D: The location of the two pollen sampling sites relative to the centre of the city (the Melbourne General Post Office). Panels A, B and D were generated using Google Maps (locations of the samplers on panel D were added manually). Panel C derives from pp. 5 of [54], with the aeroplane symbol added manually. (PDF) [file pone.0194929.s001.pdf]

**J45**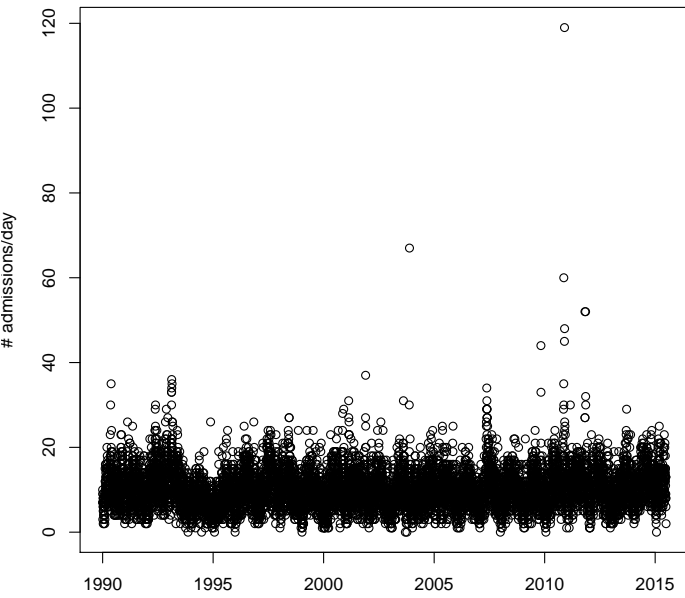**J46**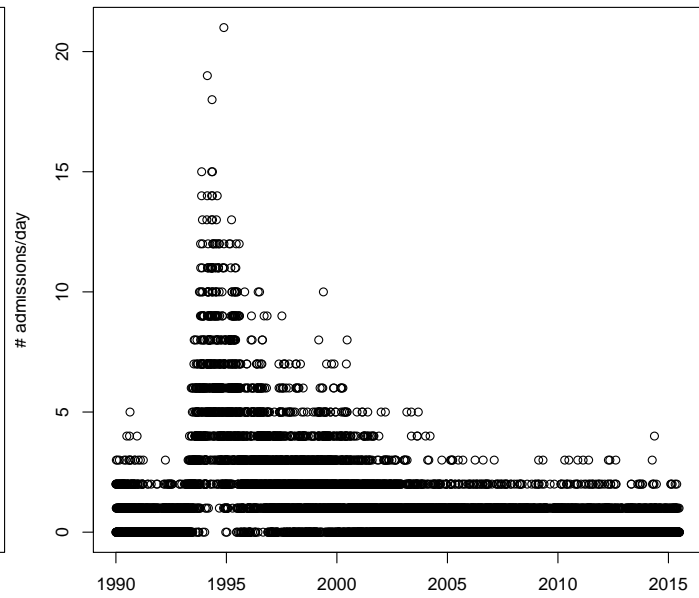**J45 + J46**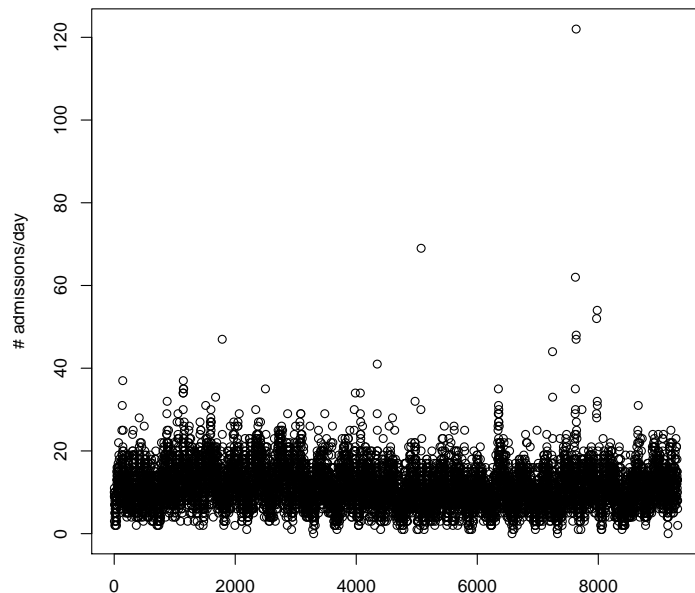

Supplement: S3 Fig — The top-left and top-right panels show daily admission numbers for admission categories J45 and J46, respectively, following the World Health Organisation’s International Classification of Diseases (ICD) 10th revision (ICD-10). The bottom panel displays the sum of the daily counts for these two diagnostic codes. The J45 category refers to asthma and includes predominantly allergic asthma, nonallergic asthma and mixed or unspecified asthma. The J45 classification excludes acute severe asthma, chronic asthmatic (obstructive) bronchitis or chronic obstructive asthma, eosinophilic asthma, lung diseases due to external agents and status asthmaticus. The J46 category refers to status asthmaticus, includeing acute severe asthma. (PDF) [file pone.0194929.s003.pdf]

**Population-normalized daily admissions**

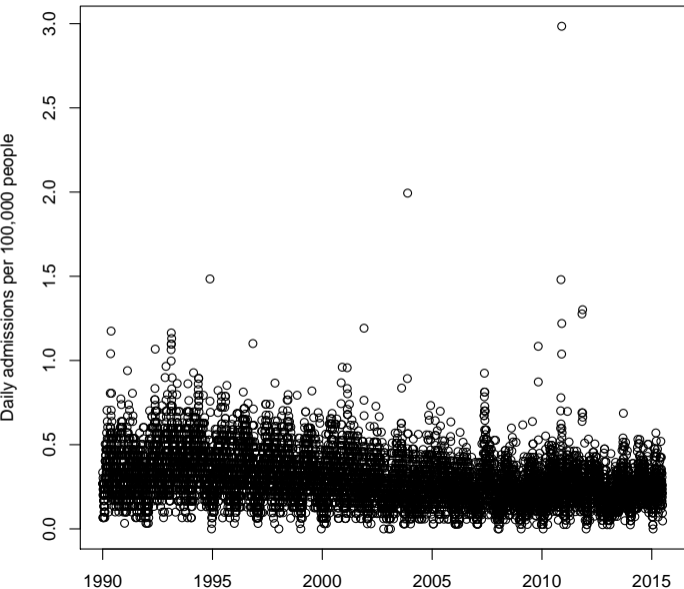

Supplement: S4 Fig — (PDF) [file pone.0194929.s004.pdf]

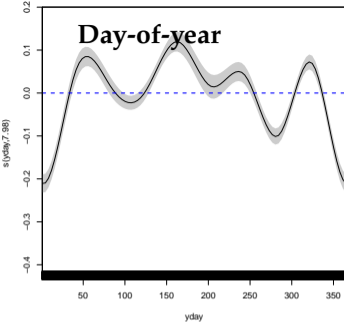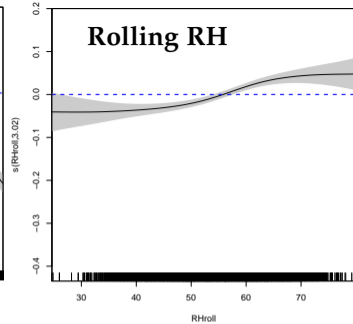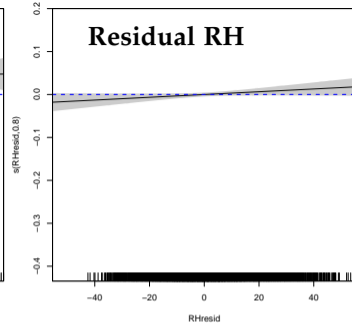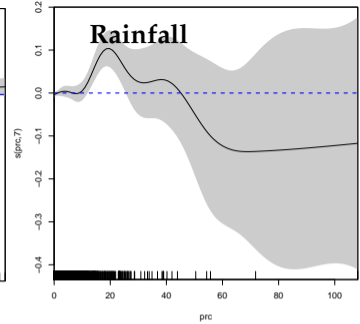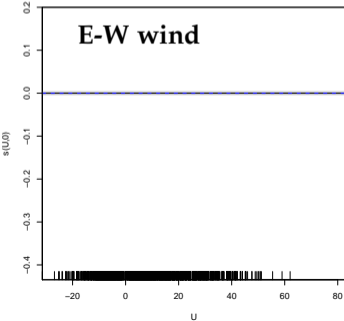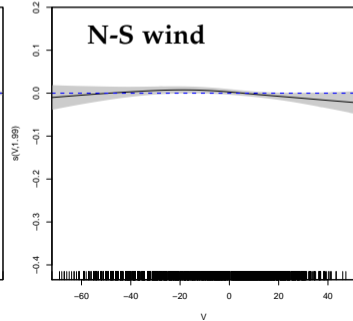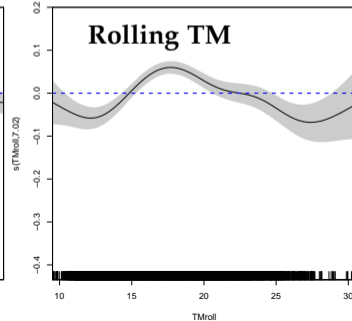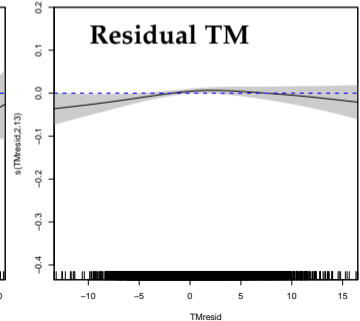

Supplement: S6 Fig — Smoothing spline fits for the non-linear terms for Model 1. The solid black line shows the estimated fit; this is surrounded by a grey area, which represents 2 standard errors above and below the estimate. These confidence bands include the uncertainty about the overall mean (thus each has an average of 0.0). The blue dashed horizontal line was drawn at y = 0.0 on each plot. The small vertical dashes from the bottom of each panel show the distribution of values of that variable. Note that the scale on the y-axis differs between the panels. To estimate the corresponding “effect size” (i.e. the additional number of admissions associated with each term in a population of 4 million), one multiplies the functions by 40. See also S4 Table. (PDF) [file pone.0194929.s006.pdf]

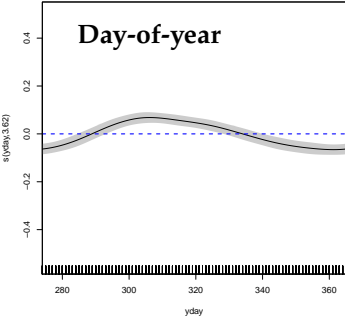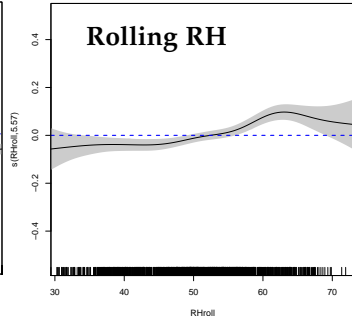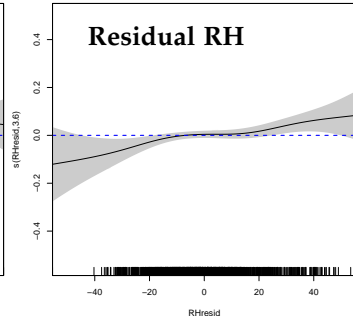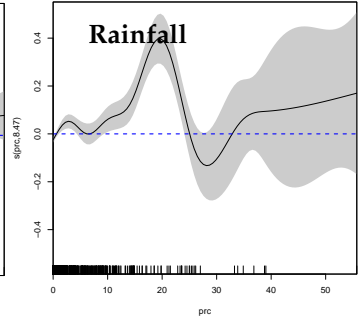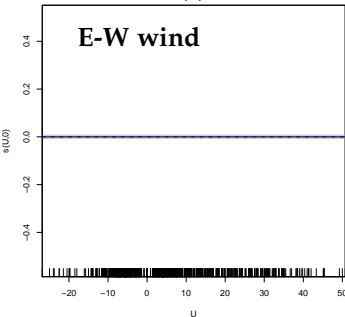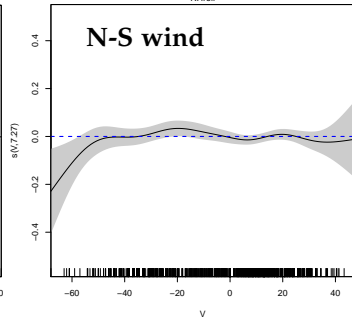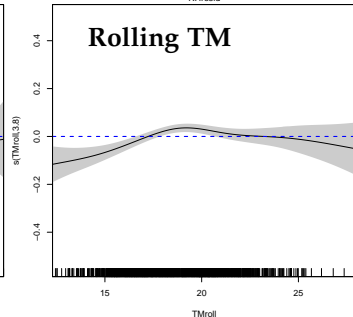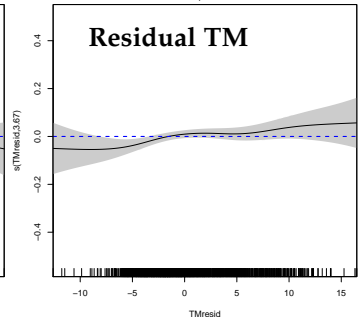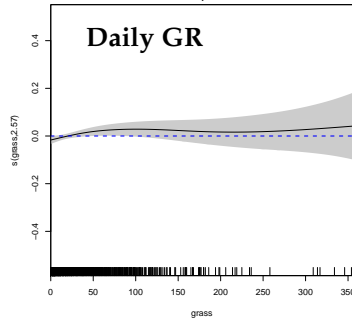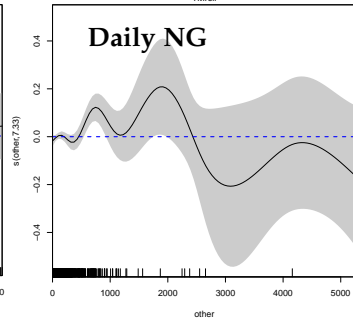

Supplement: S7 Fig — See also S5 Table and the captions of S4 Table and S6 Fig for further details. (PDF) [file pone.0194929.s007.pdf]

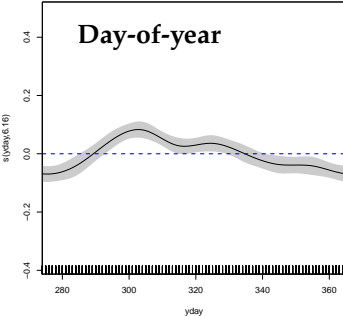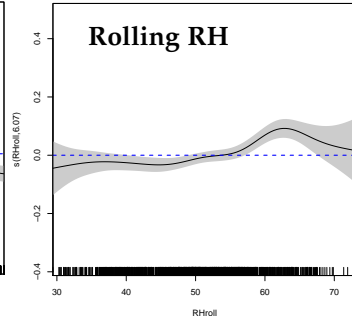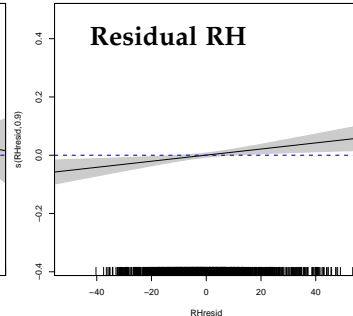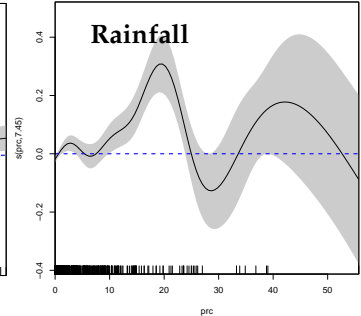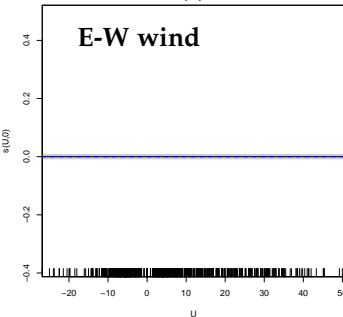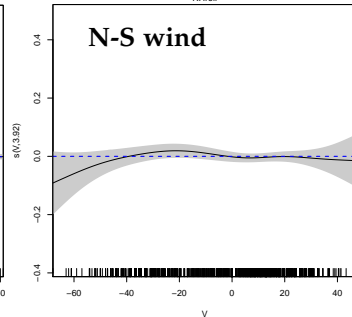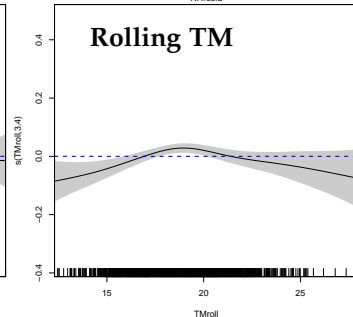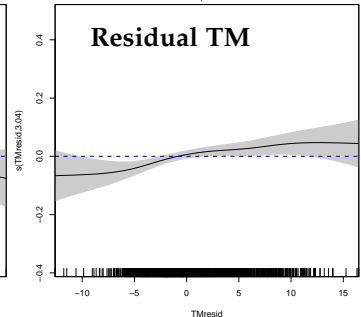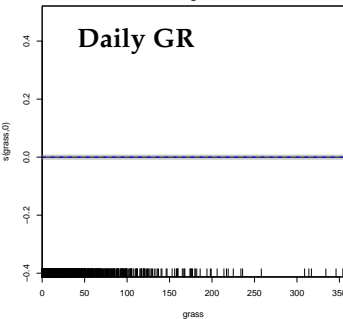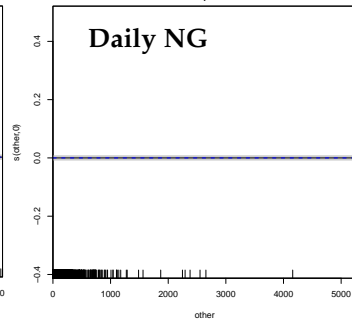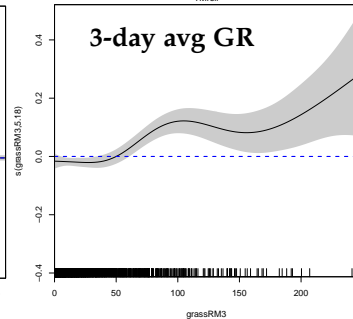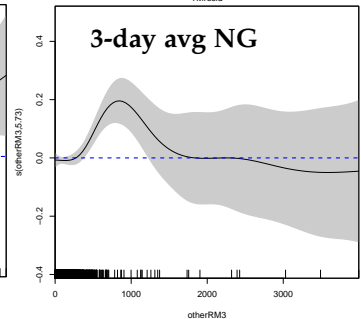

Supplement: S8 Fig — See also S6 Table and the captions of S4 Table and S6 Fig for further details. (PDF) [file pone.0194929.s008.pdf]

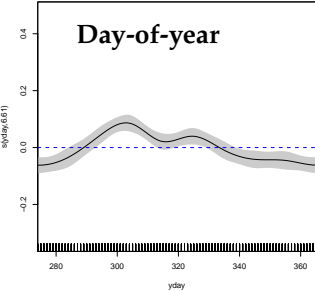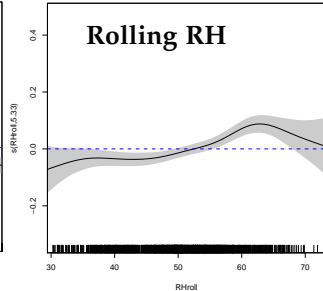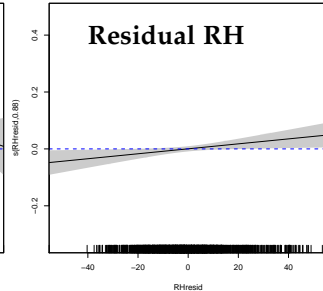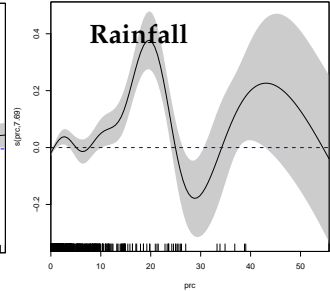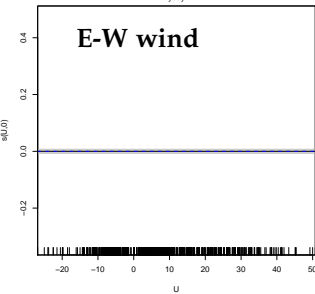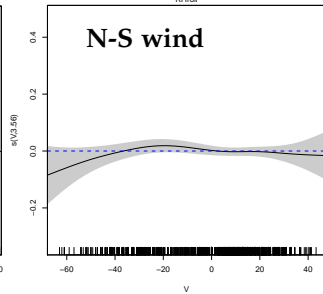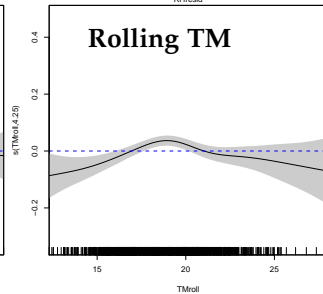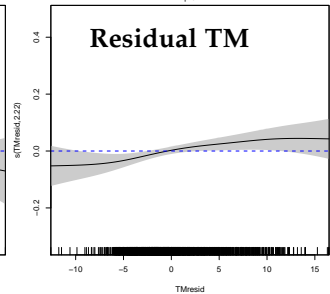

Supplement: S9 Fig — See also S7 Table and the captions of S4 Table and S6 Fig for further details. (PDF) [file pone.0194929.s009.pdf]

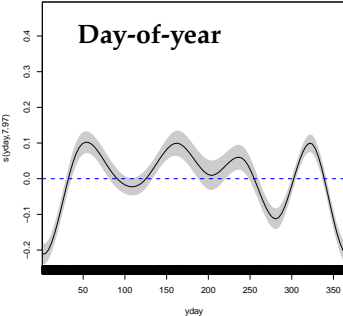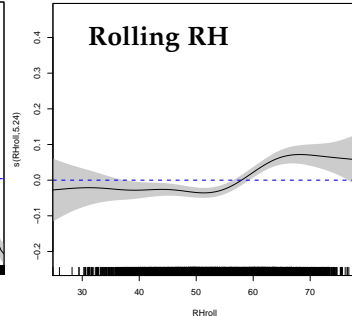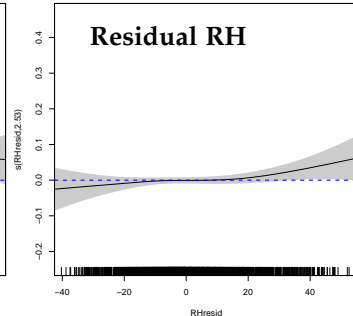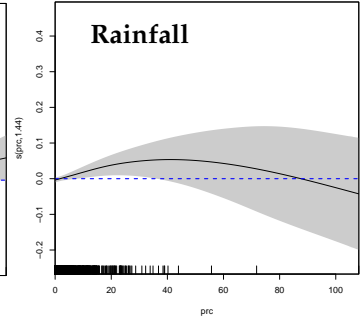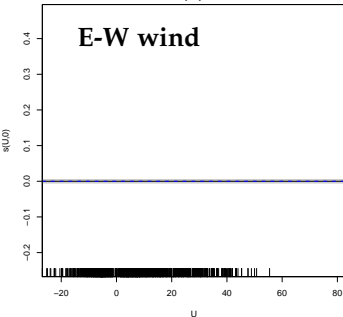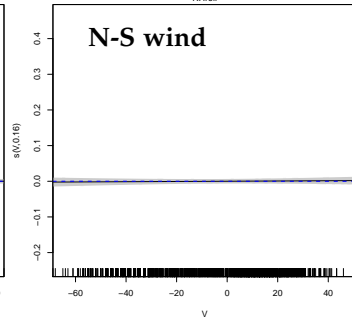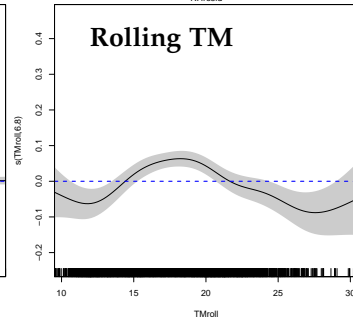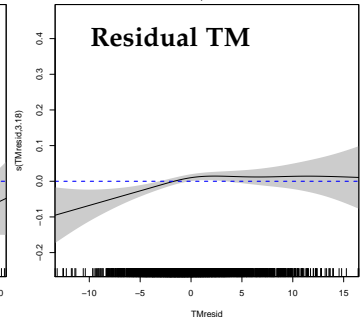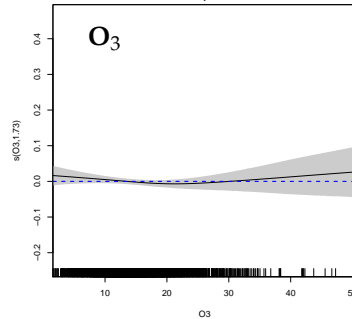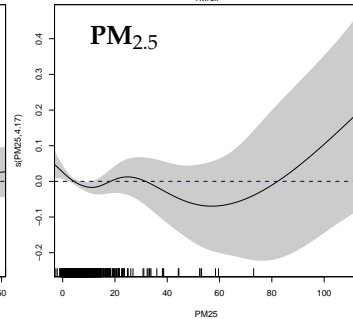

Supplement: S10 Fig — See also S8 Table and the captions of S4 Table and S6 Fig for further details. (PDF) [file pone.0194929.s010.pdf]

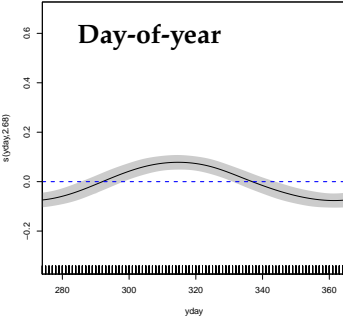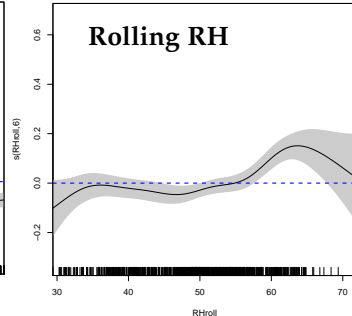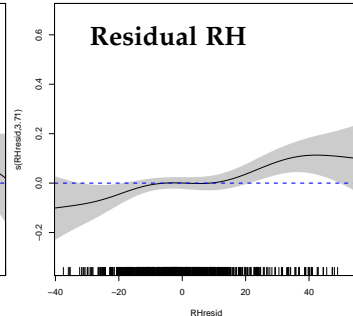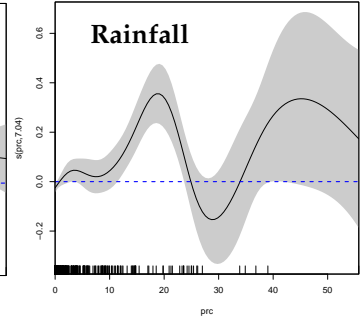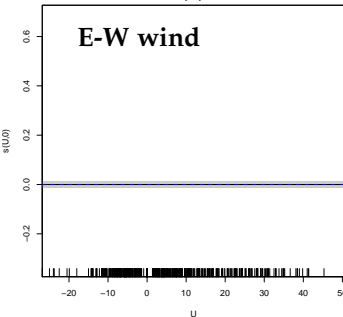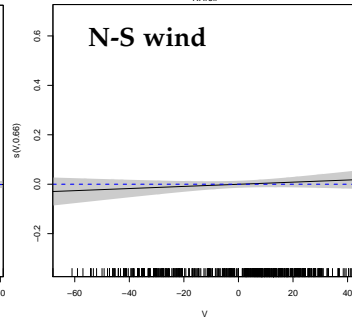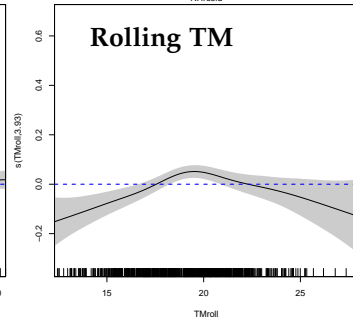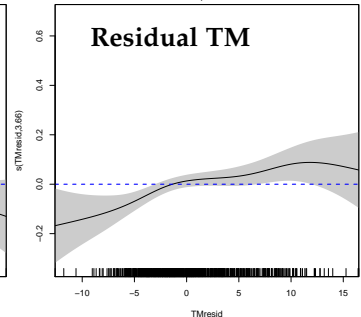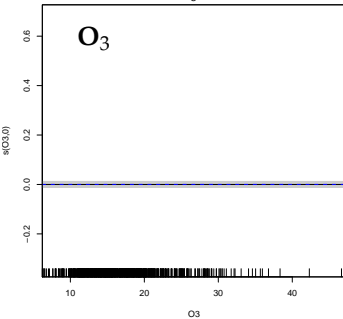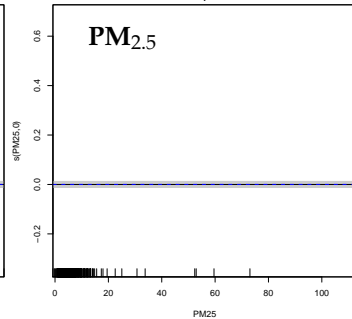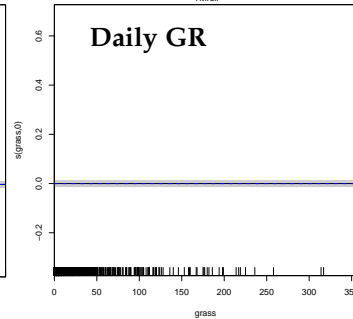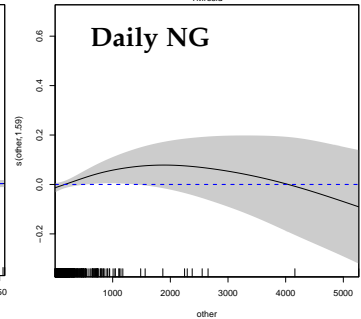

Supplement: S11 Fig — See also S9 Table and the captions of S4 Table and S6 Fig for further details. (PDF) [file pone.0194929.s011.pdf]

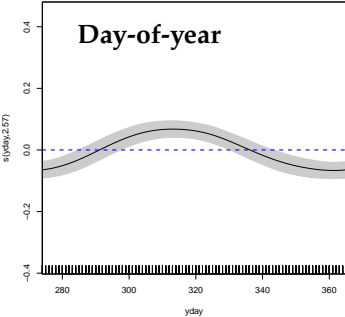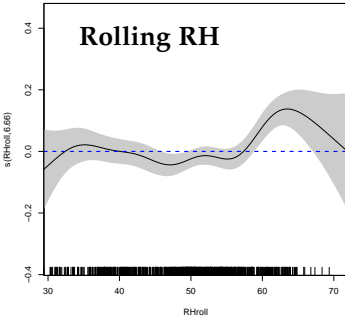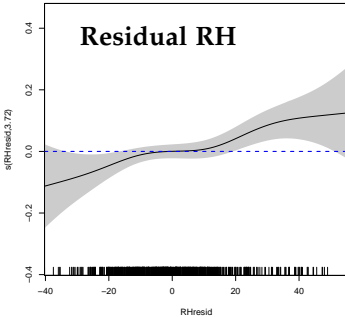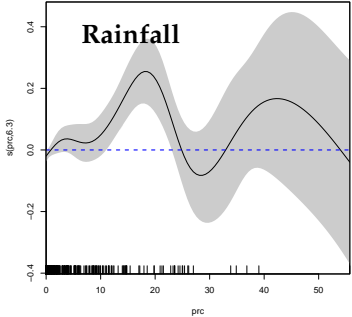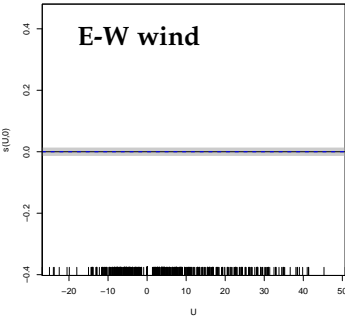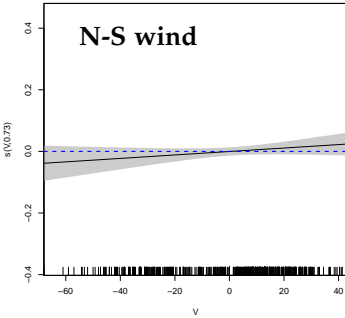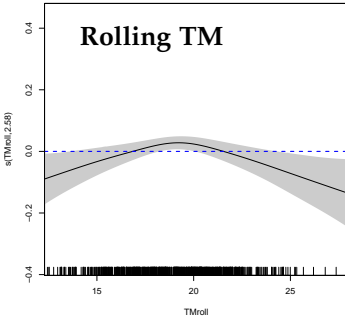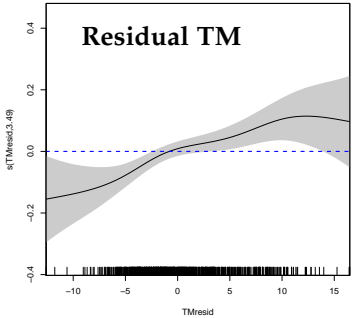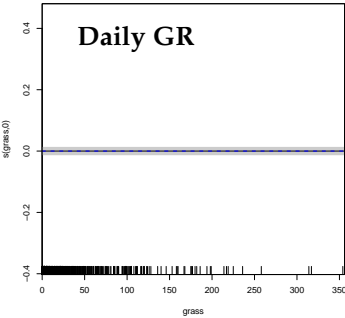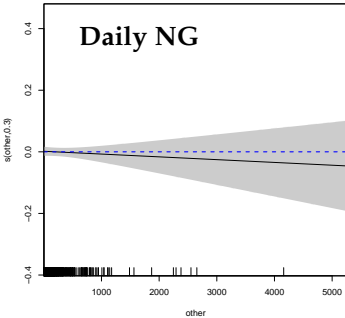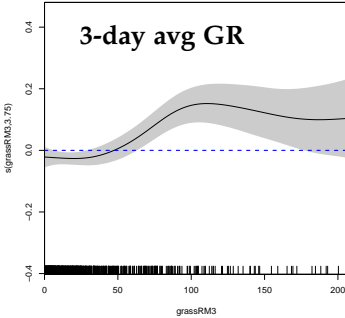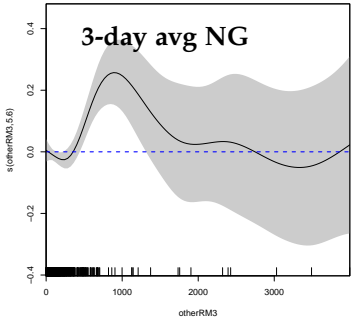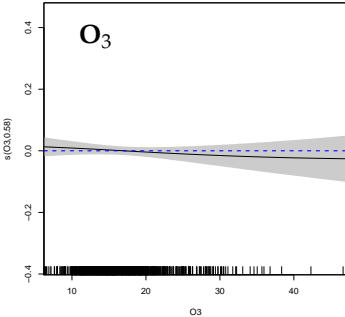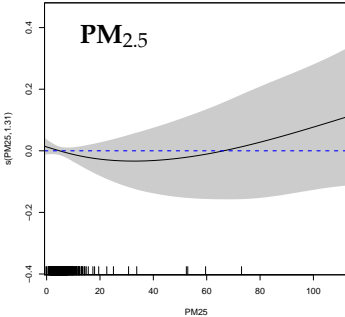

Supplement: S12 Fig — See also S10 Table and the captions of S4 Table and S6 Fig for further details. (PDF) [file pone.0194929.s012.pdf]

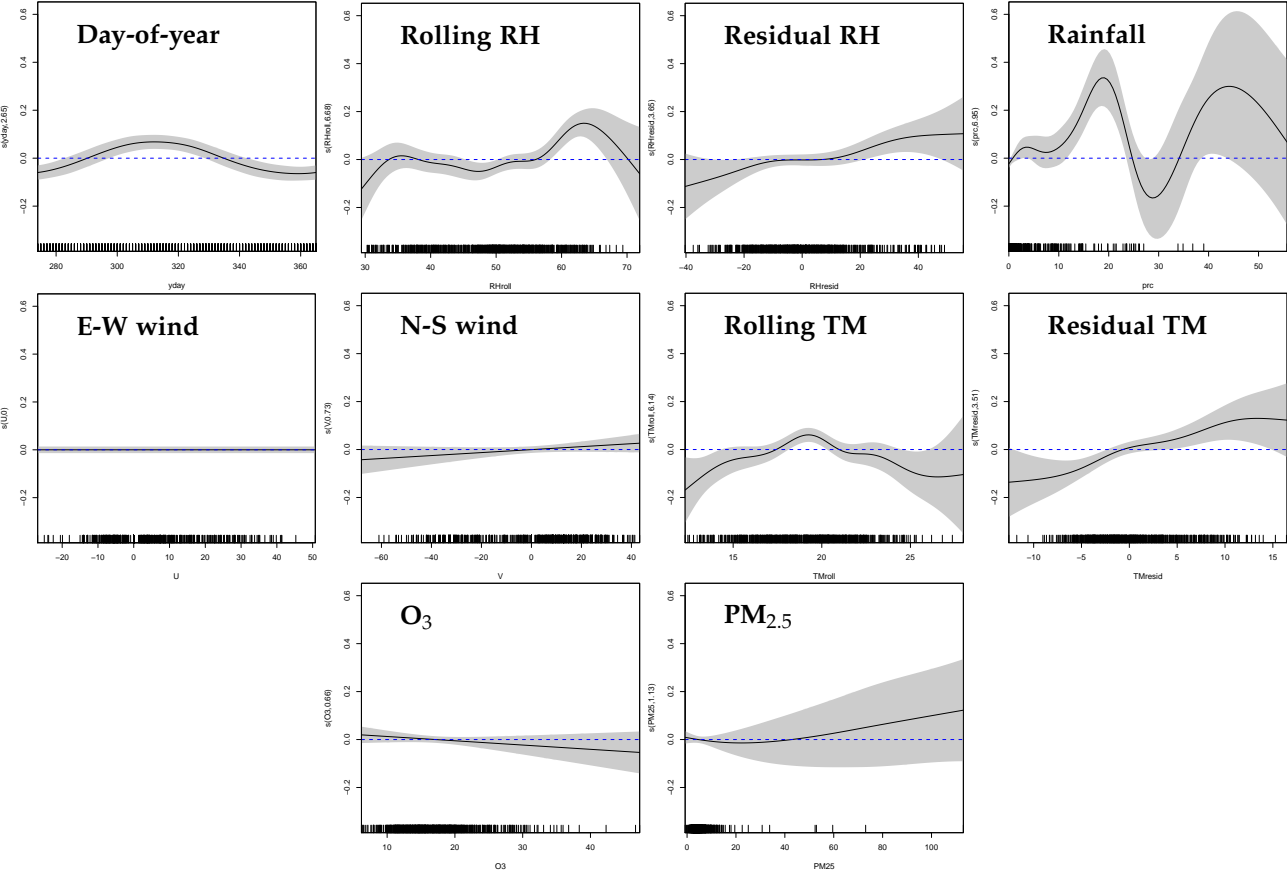

Supplement: S13 Fig — See also S11 Table and the captions of S4 Table and S6 Fig for further details. (PDF) [file pone.0194929.s013.pdf]

Normal Q-Q Plot

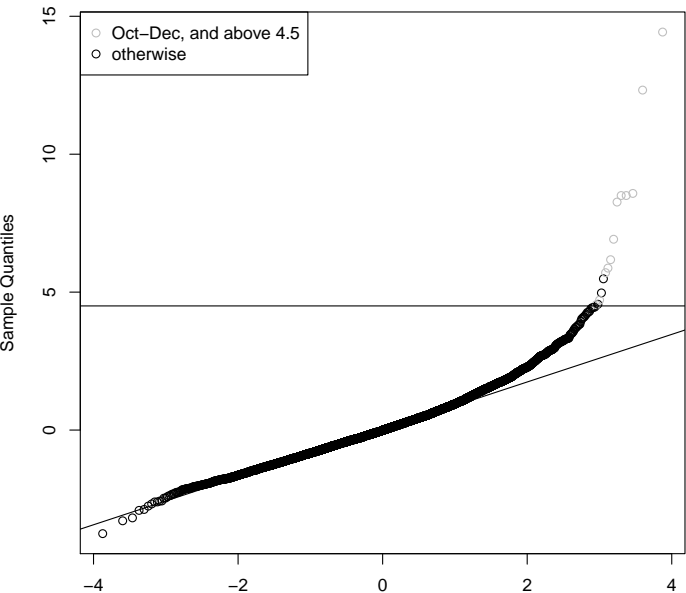

Supplement: S14 Fig — Quantile-quantile plot for the residuals normalised by the 31-day centred running mean and standard deviation (see Fig A). The horizontal line, at a value of z = 4.5, shows the threshold above which dates were labeled as “high asthma admissions days” (HAADs). Above this threshold, points were drawn in grey if they fall in the October to December period and in black at any other month of the year. (PDF) [file pone.0194929.s014.pdf]

# Mean grass pollen and number of HAADs per month

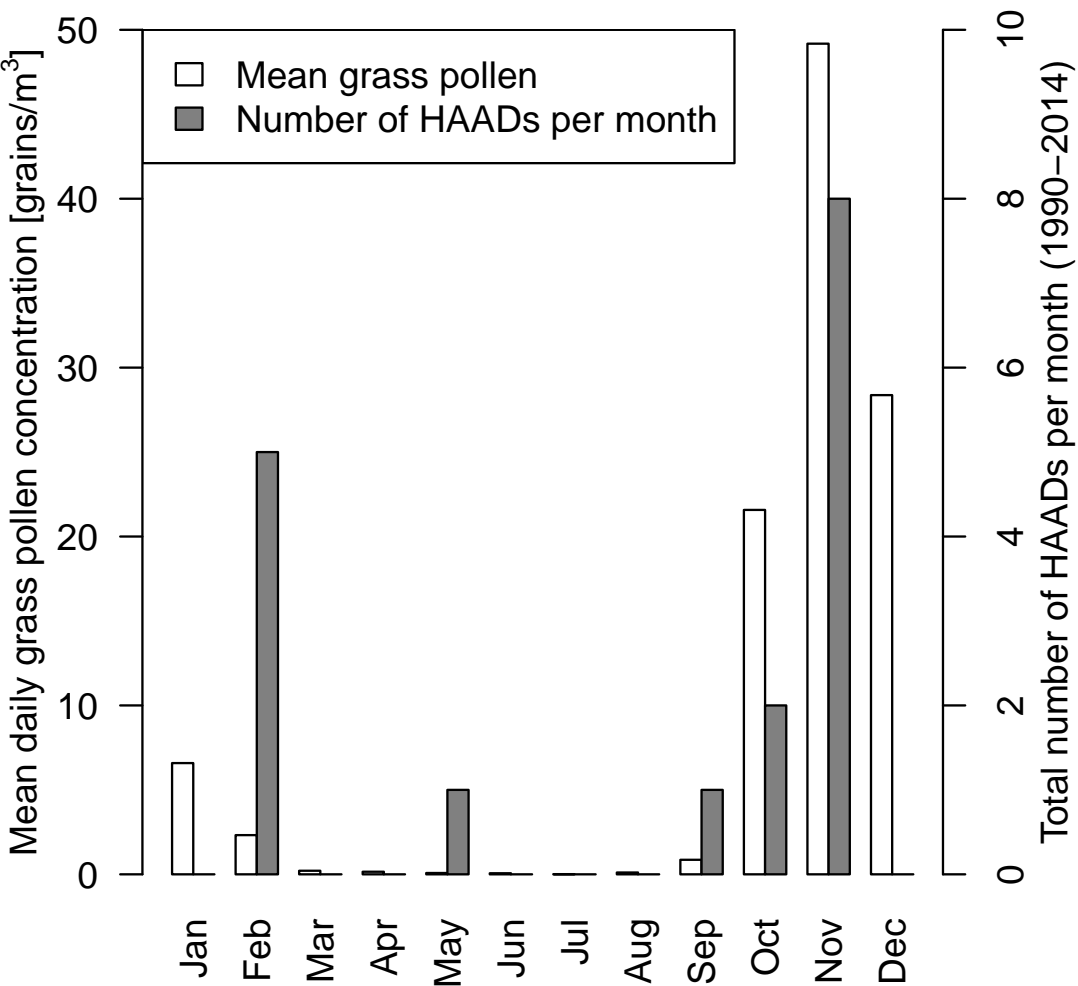

Supplement: S15 Fig — The monthly distribution of HAADs and mean monthly pollen concentration as measured in Melbourne. Grass pollen data shown here are based on Erbas et al. [61]. (PDF) [file pone.0194929.s015.pdf]

**Average admission rate per day of week and month of year**

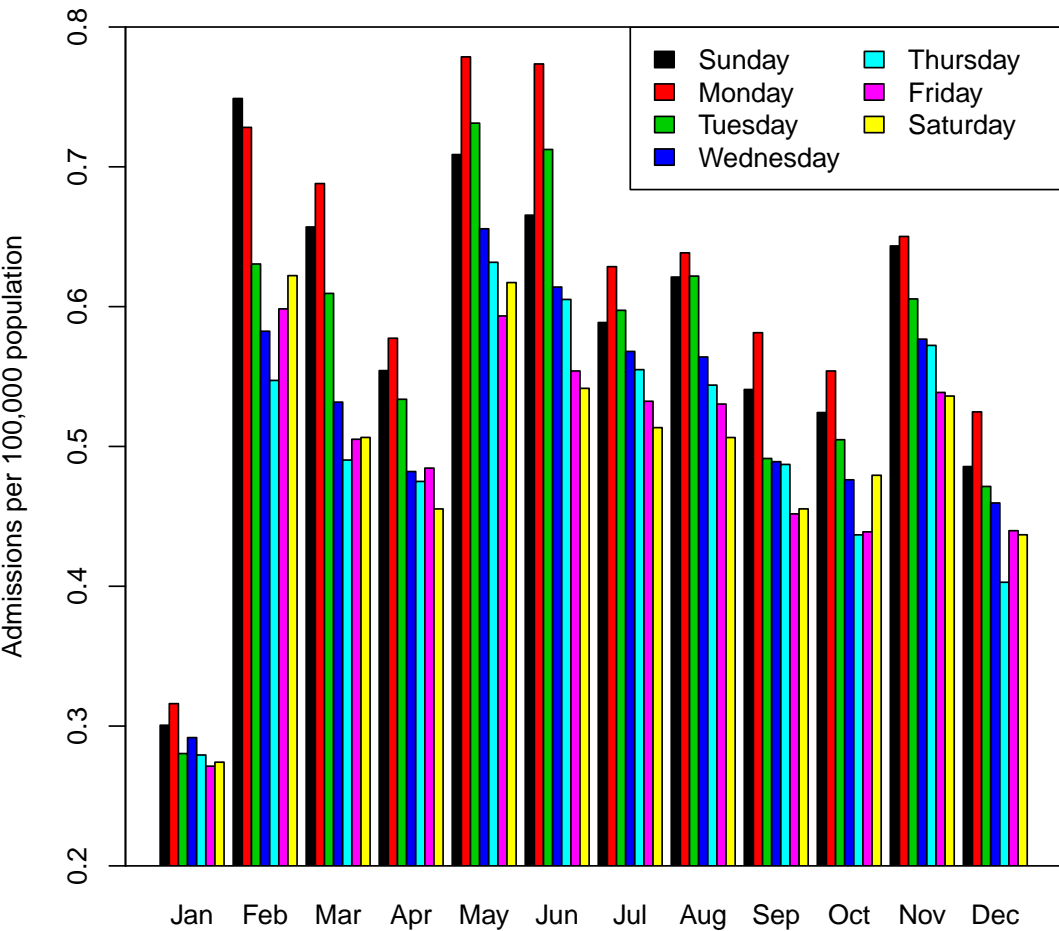

Supplement: S16 Fig — The average admissions per day-of-week, for each month of the year, across the full 1990-2015 period. (PDF) [file pone.0194929.s016.pdf]

Date = 1994-11-20

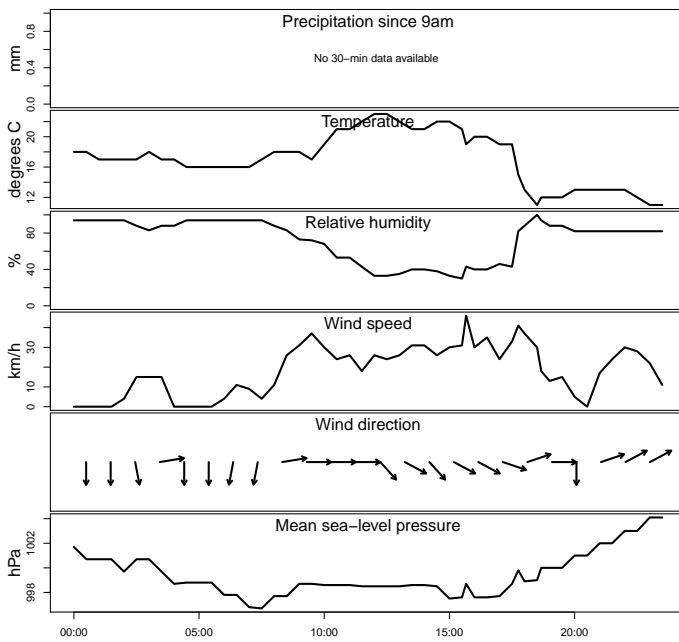

Date = 1996-11-03

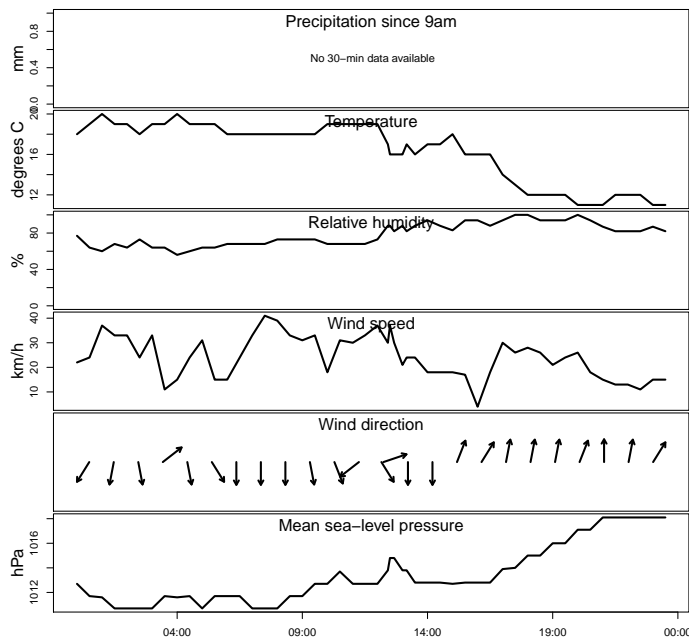

Date = 2001-11-25

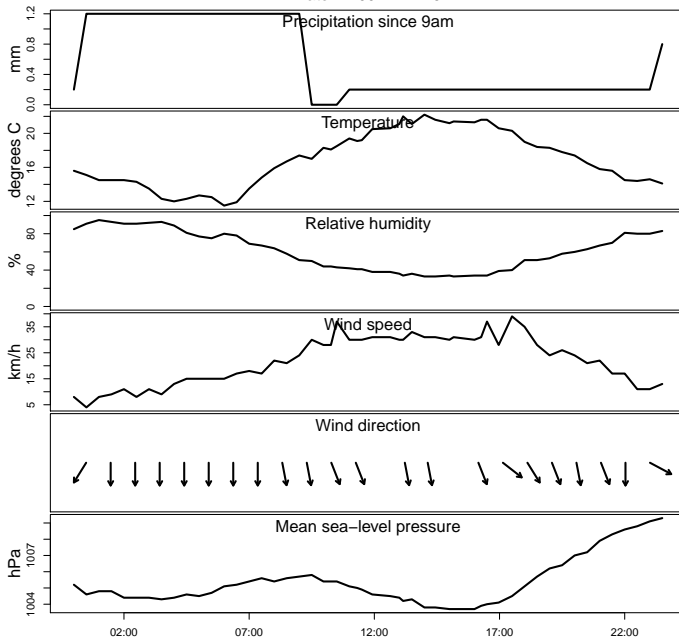

Date = 2003-11-20

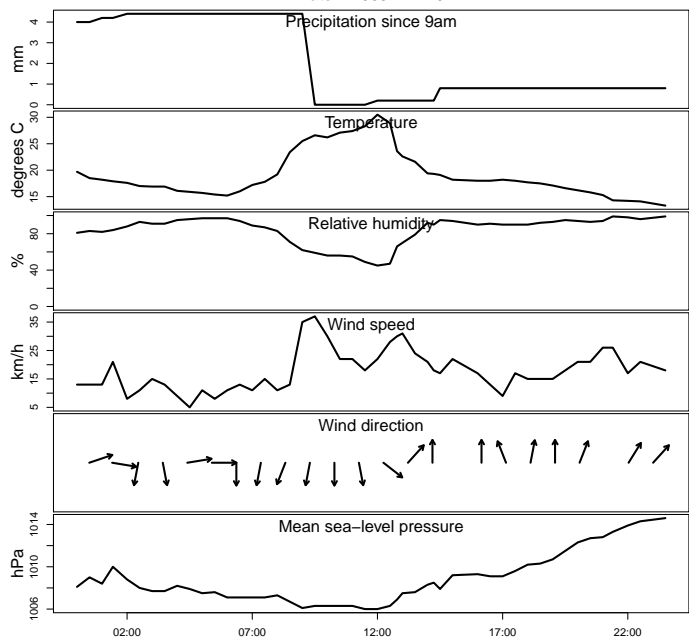

Supplement: S17 Fig — Half-hourly meteorological data recorded at Melbourne Airport (by the Bureau of Meteorology) on four high asthma admission dates in the months of October through to December (see Table 1 in the main text). The cumulative precipitation is reset at 9 am each day. Wind direction is shown when data were available at the 30 minutes past the hour. Continued in S18 and S19 Figs. (PDF) [file pone.0194929.s017.pdf]

Date = 2009-10-31

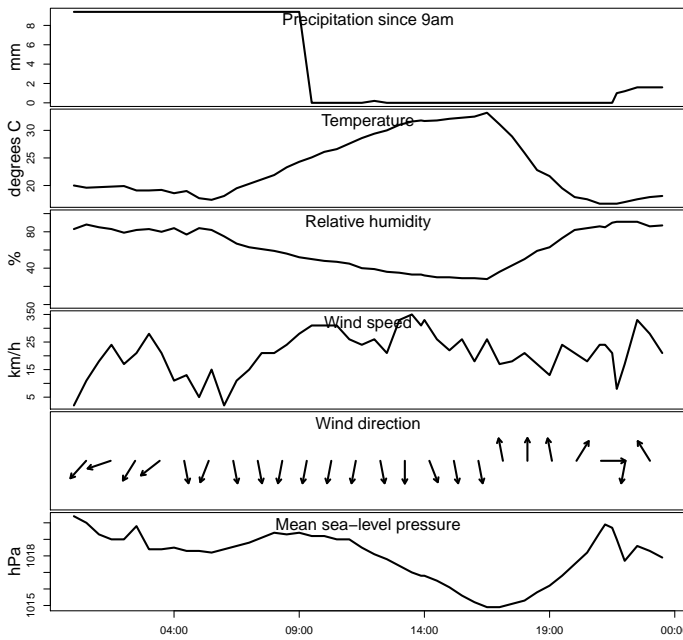

Date = 2009-11-01

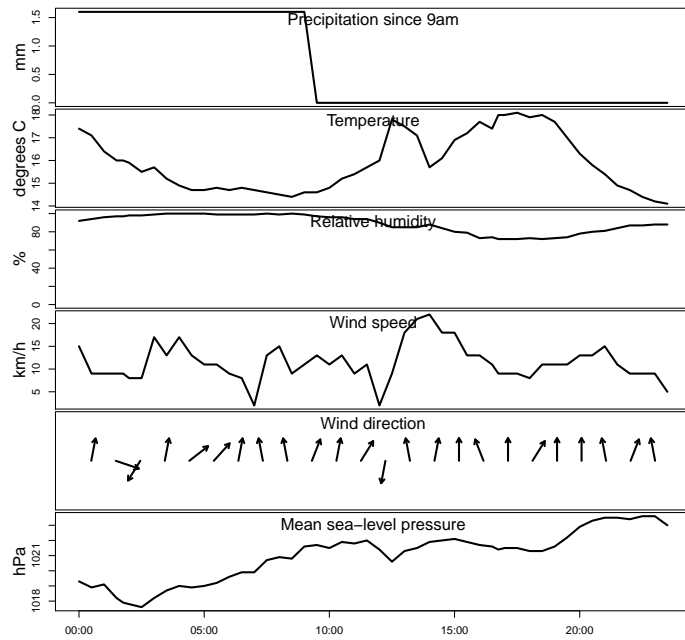

Date = 2010-11-13

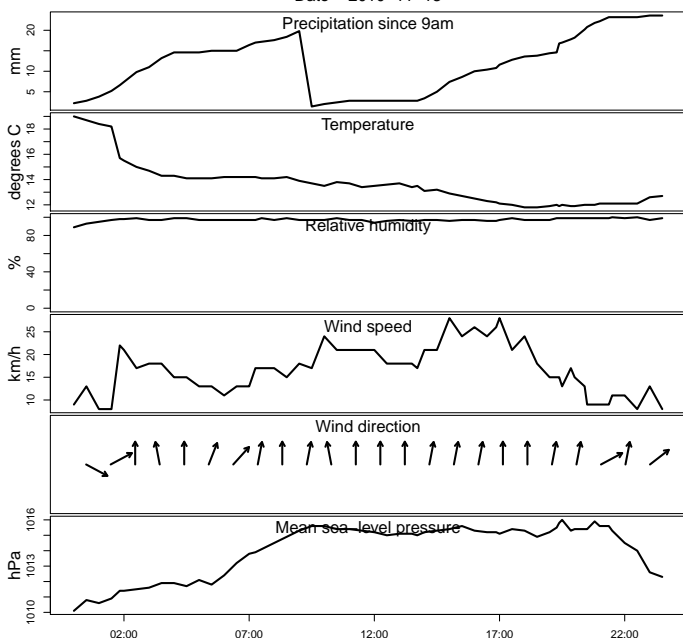

Date = 2010-11-25

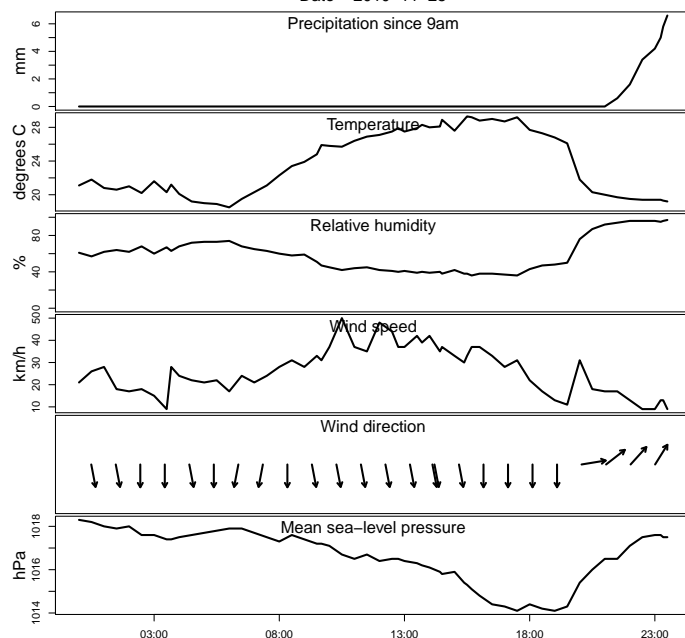

Supplement: S18 Fig — See S17 Fig for further details. (PDF) [file pone.0194929.s018.pdf]

Date = 2011-10-29

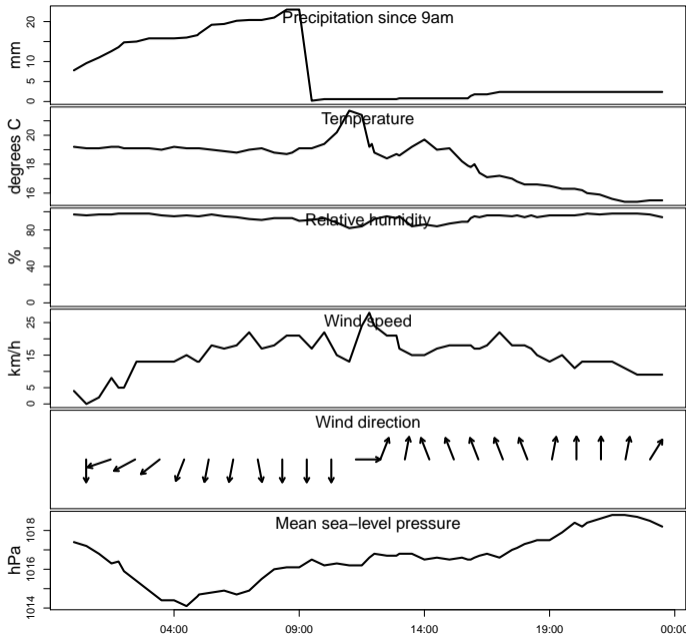

Date = 2011-11-08

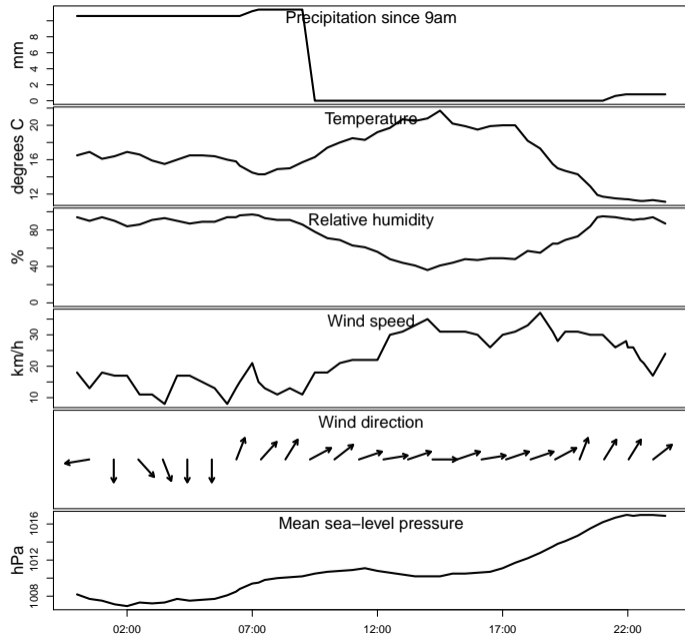

Supplement: S19 Fig — See S17 Fig for further details. (PDF) [file pone.0194929.s019.pdf]
