# Supplementary material for: Seasonal asthma in Melbourne, Australia, and some observations on the occurrence of thunderstorm asthma and its predictability
Source: PLoS One. 2018 Apr 12;13(4):e0194929. doi: 10.1371/journal.pone.0194929 (PMC5896915; doi:10.1371/journal.pone.0194929)
Supplement: S2 Fig — Sites are shown relative to the Melbourne Central Business Distict. The Alphington site is indicated. See S1 Fig for the broader geographical context. (PDF) [file pone.0194929.s002.pdf]

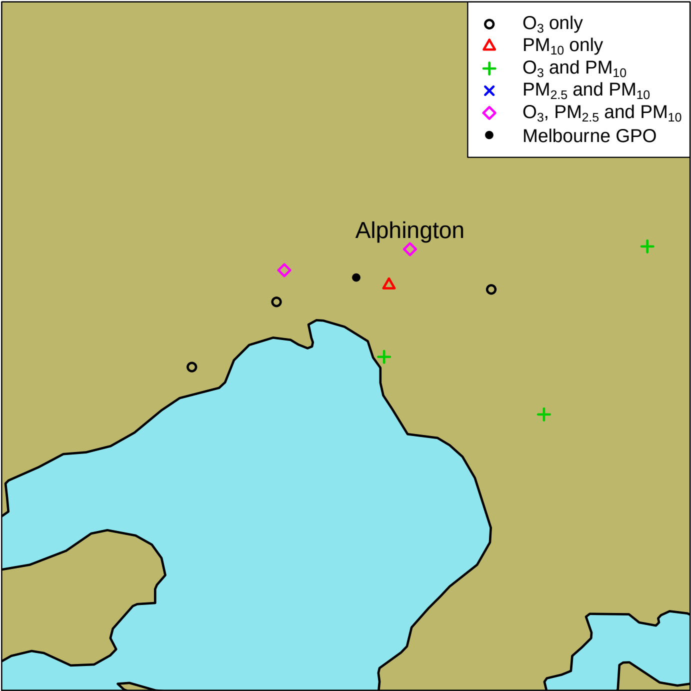
O_3 only
PM_{10} only
O_3 and PM_{10}
$PM_{2.5}$ and PM_{10}
O_3, $PM_{2.5}$ and PM_{10}
Melbourne GPO
Alphington
