# Supplementary material for: Seasonal asthma in Melbourne, Australia, and some observations on the occurrence of thunderstorm asthma and its predictability
Source: PLoS One. 2018 Apr 12;13(4):e0194929. doi: 10.1371/journal.pone.0194929 (PMC5896915; doi:10.1371/journal.pone.0194929)
Supplement: S5 Fig — Residuals in the admissions time-series. A: residuals normalised by the 31-day centred running mean and standard deviation. B: residuals normalised by the annual mean and standard deviation. C: population-normalised residuals minus the seasonal component (Fig 1 in the main text). (PDF) [file pone.0194929.s005.pdf]

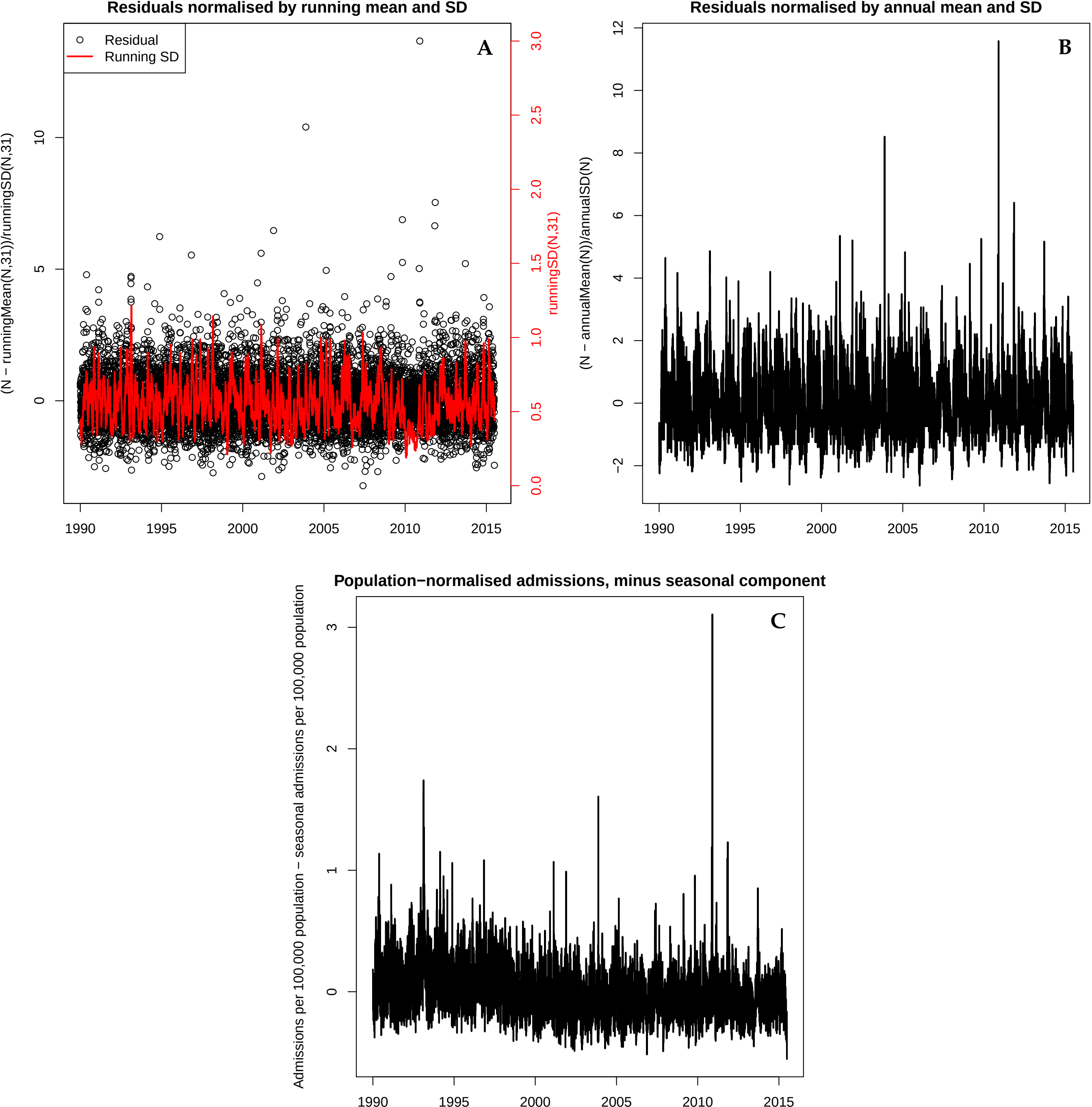
Residuals normalised by running mean and SD
Residual
Running SD
A
(N − runningMean(N,31))/runningSD(N,31)
runningSD(N,31)
0.0
0.5
1.0
1.5
2.0
2.5
3.0
0
5
10
1990
1995
2000
2005
2010
2015
Residuals normalised by annual mean and SD
B
(N − annualMean(N))/annualSD(N)
−2
0
2
4
6
8
10
12
1990
1995
2000
2005
2010
2015
Population−normalised admissions, minus seasonal component
C
Admissions per 100,000 population − seasonal admissions per 100,000 population
0
1
2
3
1990
1995
2000
2005
2010
2015
