# Supplementary material for: Seasonal asthma in Melbourne, Australia, and some observations on the occurrence of thunderstorm asthma and its predictability
Source: PLoS One. 2018 Apr 12;13(4):e0194929. doi: 10.1371/journal.pone.0194929 (PMC5896915; doi:10.1371/journal.pone.0194929)
Supplement: S1 Table — The seven thunderstorm metrics considered, compared using the Equitable Threat Score (ETS), a measure of the correspondence between the classification of thunderstorm events, corrected for chance agreement. A value of 1.0 indicates perfect agreement, and the statistic ranges from −1/3 to 1.0. The column on the right-hand side show the total number of events indicated during the three-year period. (PDF) [file pone.0194929.s020.pdf]

	GPATS > 2	Gridded > 2	METAR	Thunder	CAPE > 5	High prec.	TS archive	Total
GPATS > 2	1.00	0.54	0.49	0.375	0.166	0.18	0.207	87
Gridded > 2	0.54	1.00	0.44	0.237	0.180	0.16	0.166	138
METAR	0.49	0.44	1.00	0.354	0.123	0.18	0.158	99
Thunder	0.37	0.24	0.35	1.000	0.095	0.15	0.213	43
CAPE > 5	0.17	0.18	0.12	0.095	1.000	0.07	0.063	178
High prec.	0.18	0.16	0.18	0.150	0.070	1.00	0.130	59
TS archive	0.21	0.17	0.16	0.213	0.063	0.13	1.000	27
