# Supplementary material for: Seasonal asthma in Melbourne, Australia, and some observations on the occurrence of thunderstorm asthma and its predictability
Source: PLoS One. 2018 Apr 12;13(4):e0194929. doi: 10.1371/journal.pone.0194929 (PMC5896915; doi:10.1371/journal.pone.0194929)
Supplement: S2 Table — For each variable, details are provided of their respective spatial and temporal resolutions. Abbreviations: BoM = Australian Bureau of Meteorology, MPC = Melbourne Pollen Count. Variable types are coded as follows: P = predictor, O = outcome, N = numerical, B = binary, C = categorical. The seasonal effect (the derived variable in the last row) is shown in Fig 1A in the main text. (PDF) [file pone.0194929.s021.pdf]

Variable	Type	Time	Abbreviation
North-south wind-speed	N,P	Midday	NS
East-west wind-speed	N,P	Midday	EW
Precipitation	N,P	9am-midnight	PR
Temperature	N,P	Midday	TM
Relative humidity	N,P	Midday	RH
Normalised admissions	N,O	00:00h to 23:59h	NA
Mean grass pollen	N,P	4pm previous day to 4pm this day	GR
Mean non-grass pollen	N,P	4pm previous day to 4pm this day	NG
Thunderstorm observed	B,P	00:00h to 23:59h	TS
Weekday	C,P	—	WK
Seasonal effect	N,P	Daily	YD
Ozone (O_3)	N,P	Daily average	O_3
Aerosols ($PM_{2.5}$)	N,P	Daily average	$PM_{2.5}$
**Variable**	**Unit**	**Location**	**Data source**
North-south wind-speed	km/h	Melbourne Airport	BoM
East-west wind-speed	km/h	Melbourne Airport	BoM
Precipitation	mm	Melbourne Airport	BoM
Temperature	Degrees C	Melbourne Airport	BoM
Relative humidity	%	Melbourne Airport	BoM
Normalised admissions	Per 100,000 population	Melbourne metro	HosData
Mean grass pollen	Grains/m^3	Parkville	MPC
Mean non-grass pollen	Grains/m^3	Parkville	MPC
Thunderstorm observed	Yes/No	In the vicinity of Melbourne Airport	BoM
Weekday	—	—	derived
Seasonal effect	Per 100,000 population	Melbourne metro	derived
Ozone (O_3)	Parts per billion	Average over sites	Vic. EPA
Aerosols ($PM_{2.5}$)	$\mu g/m^3$	Alphington	Vic. EPA
