# Supplementary material for: Seasonal asthma in Melbourne, Australia, and some observations on the occurrence of thunderstorm asthma and its predictability
Source: PLoS One. 2018 Apr 12;13(4):e0194929. doi: 10.1371/journal.pone.0194929 (PMC5896915; doi:10.1371/journal.pone.0194929)
Supplement: S3 Table — Summary of regression models considered. Abbreviations: Step = use step-wise variable selection?, YD = seasonal or day-of-year effect, RH = relative humidity, TM = temperature, PR = precipitation, NS = north-south wind component, EW = east-wst wind component, TS = thunderstorm, GR = grass pollen, NG = non-grass pollen, WK = weekday, * = decomposed into a rolling (backward-looking) 14-day mean and the daily deviation from this rolling mean, † = consider lagged variable over different times, A × B = interactions between variables A and B, Y = yes, N = no. When creating lagged variables for the pollen time-series, two variables were used in the regression (per pollen type—grass or non-grass): the value on the day of interest, and th means of the 3 days prior to the day of interest (i.e. not including the value of the day of interest). (PDF) [file pone.0194929.s022.pdf]

ID	Step	Non-linear predictor variables	Categorical predictors
1	N	YD, RH*, TM*, PR, NS, EW	TS, WK
2	N	YD, RH*, TM*, PR, NS, EW, GR, NG,	TS, WK
3	N	YD, RH*, TM*, PR, NS, EW, GR†, NG†,	TS, WK
4	Y	YD, RH*, TM*, PR, NS, EW, TS × (GR†, NG†)	WK
5	N	YD, RH*, TM*, PR, NS, EW, O_3, $PM_{2.5}$	TS, WK
6	N	YD, RH*, TM*, PR, NS, EW, GR, NG, O_3, $PM_{2.5}$	TS, WK
7	N	YD, RH*, TM*, PR, NS, EW, GR†, NG†, O_3, $PM_{2.5}$	TS, WK
8	Y	YD, RH*, TM*, PR, NS, EW, TS × (GR†, NG†), WK, O_3, $PM_{2.5}$	WK
**ID**	**Period**	**notes**	
1	Jan-Dec, '00-15	No pollen, use full year, no AQ data	
2	Oct-Dec, '00-15	With pollen, only when pollen is available, no AQ data	
3	Oct-Dec, '00-15	With pollen and lagged pollen, no AQ data	
4	Oct-Dec, '00-15	Step-wise variable selection with pollen-TS interactions, no AQ data	
5	Jan-Dec, '03-14	No pollen, full year, with AQ data	
6	Oct-Dec, '03-14	With pollen & AQ data, only when pollen is available	
7	Oct-Dec, '03-14	With pollen and lagged pollen, with AQ data	
8	Oct-Dec, '03-14	Step-wise variable selection with pollen-TS interactions, with AQ data	
