# Supplementary material for: Seasonal asthma in Melbourne, Australia, and some observations on the occurrence of thunderstorm asthma and its predictability
Source: PLoS One. 2018 Apr 12;13(4):e0194929. doi: 10.1371/journal.pone.0194929 (PMC5896915; doi:10.1371/journal.pone.0194929)
Supplement: S4 Table — Summary of the fit for Model 1 (see S3 Table). The upper part of the table corresponds to the linear, binary or categorical terms whereas the lower half of the table provides information about fit of continuous variables that were allowed to vary non-linearly. The first column in both parts of the table gives the name of each term. Abbreviations used in the model summaries: yday = day-of-year effect; RH = relative humidity; TM = temperature; PR = precipitation; NS = north-south wind component; EW = east-wst wind component; TS = thunderstorm; GR = grass pollen; NG = non-grass pollen; WK = weekday; A:B = the interaction term between binary variable A and numerical variable B (set to zero when A is false); subscripts “rl” and “dv” = the rolling, backward-looking 14-day mean and the daily deviation from this rolling mean (respectively); terms WKM, WKTu, WKW, WKTh, WKF, WKS = the day-of-week effect (for Monday through to Saturday, respectively), relative to Sunday (positive values mean higher than the effect for Sunday); the EDF represents the estimated number of degrees of freedom for the non-linear terms. If the EDF is equal to 1.0, then the term shows no evidence of non-linearity; if the EDF is equal to 0.0, then the term has been dropped via shrinkage (implicit model selection). In the upper half of the table, the t-statistic and the associated p-value corresponds to a test for whether the parameter is non-zero. The “Effect size” column displays an estimate of the number of daily admissions, in a population of 4.0 million, associated with each term (see Appendix 2); the associated confidence interval accounts only for uncertainty in the regression coefficient, and does not address the range of the predictor variable. The lower part of the table shows information about the non-linear terms. The F-statistic and the associated p-value corresponds to a test for whether all the coefficients associated with this term are zero. (PDF) [file pone.0194929.s023.pdf]

	t value	$\Pr(>\|t\|)$	Effect size (95% CI)
(Intercept)	81.222	0.000	19.40 (18.92, 19.87)
TS	4.707	0.000	1.55 (0.89, 2.21)
WK_M	1.671	0.095	0.56 (-0.11, 1.23)
WK_{Tu}	-2.960	0.003	-0.99 (-1.66, -0.32)
WK_{We}	-6.838	0.000	-2.30 (-2.97, -1.62)
WK_{Th}	-8.622	0.000	-2.90 (-3.57, -2.23)
WK_F	-9.478	0.000	-3.19 (-3.86, -2.52)
WK_S	-9.071	0.000	-3.04 (-3.71, -2.37)
	F value	$\Pr(> F)$	EDF
yday	121.355	0.000	7.983
RH_{rl}	5.646	0.000	3.019
RH_{dv}	0.347	0.046	0.804
PR	3.665	0.000	7.002
EW	0.000	0.519	0.000
NS	0.660	0.031	1.987
TM_{rl}	12.373	0.000	7.019
TM_{dv}	0.832	0.012	2.130
