# Supplementary material for: Seasonal asthma in Melbourne, Australia, and some observations on the occurrence of thunderstorm asthma and its predictability
Source: PLoS One. 2018 Apr 12;13(4):e0194929. doi: 10.1371/journal.pone.0194929 (PMC5896915; doi:10.1371/journal.pone.0194929)
Supplement: S5 Table — Summary of the fit for Model 2 (see S3 Table). See the caption of S4 Table for further details. (PDF) [file pone.0194929.s024.pdf]

	t value	$\Pr(>\|t\|)$	Effect size
(Intercept)	35.528	0.000	18.34 (17.30, 19.37)
TS	2.443	0.015	1.43 (0.26, 2.61)
WK_M	-0.228	0.820	-0.16 (-1.58, 1.26)
WK_{Tu}	-1.335	0.182	-0.95 (-2.37, 0.47)
WK_{We}	-3.000	0.003	-2.14 (-3.57, -0.71)
WK_{Th}	-3.552	0.000	-2.56 (-4.00, -1.12)
WK_F	-4.262	0.000	-3.06 (-4.49, -1.62)
WK_S	-2.411	0.016	-1.72 (-3.16, -0.29)
	F value	$\Pr(> F)$	EDF
yday	10.438	0.000	3.619
RH_{rl}	5.492	0.000	5.573
RH_{dv}	1.461	0.003	3.602
PR	10.599	0.000	8.466
EW	0.000	0.437	0.000
NS	1.658	0.032	7.267
TM_{rl}	3.610	0.000	3.804
TM_{dv}	1.167	0.013	3.666
GR	0.693	0.054	2.572
NG	2.906	0.000	7.333
