# Supplementary material for: Seasonal asthma in Melbourne, Australia, and some observations on the occurrence of thunderstorm asthma and its predictability
Source: PLoS One. 2018 Apr 12;13(4):e0194929. doi: 10.1371/journal.pone.0194929 (PMC5896915; doi:10.1371/journal.pone.0194929)
Supplement: S6 Table — Summary of the fit for Model 3 (see S3 Table). See the caption of S4 Table for further details. (PDF) [file pone.0194929.s025.pdf]

	t value	$\Pr(>\|t\|)$	Effect size
(Intercept)	36.551	0.000	18.27 (17.27, 19.27)
TS	2.786	0.005	1.58 (0.44, 2.71)
WK_M	0.056	0.955	0.04 (-1.33, 1.41)
WK_{Tu}	-1.263	0.207	-0.87 (-2.25, 0.51)
WK_{We}	-2.853	0.004	-1.97 (-3.36, -0.59)
WK_{Th}	-3.506	0.000	-2.44 (-3.84, -1.05)
WK_F	-4.471	0.000	-3.11 (-4.50, -1.72)
WK_S	-2.744	0.006	-1.90 (-3.28, -0.51)
	F value	$\Pr(> F)$	EDF
yday	10.460	0.000	6.161
RH_{rl}	4.608	0.000	6.066
RH_{dv}	0.827	0.004	0.897
PR	7.464	0.000	7.449
EW	0.000	1.000	0.000
NS	0.711	0.135	3.919
TM_{rl}	2.549	0.000	3.403
TM_{dv}	1.462	0.002	3.045
GR	0.000	0.854	0.000
NG	0.000	0.508	0.000
GR_{m3}	6.582	0.000	5.182
NG_{m3}	4.120	0.000	5.727
