# Supplementary material for: Seasonal asthma in Melbourne, Australia, and some observations on the occurrence of thunderstorm asthma and its predictability
Source: PLoS One. 2018 Apr 12;13(4):e0194929. doi: 10.1371/journal.pone.0194929 (PMC5896915; doi:10.1371/journal.pone.0194929)
Supplement: S7 Table — Summary of the fit for Model 4 (see S3 Table). See the caption of S4 Table for further details. (PDF) [file pone.0194929.s026.pdf]

	t value	$\Pr(>\lvert t\rvert)$	Effect size
(Intercept)	29.985	0.000	17.04 (15.90, 18.18)
WK_M	0.031	0.976	0.02 (-1.37, 1.41)
WK_{Tu}	-1.450	0.147	-1.01 (-2.41, 0.38)
WK_{We}	-3.024	0.003	-2.12 (-3.52, -0.72)
WK_{Th}	-3.430	0.001	-2.41 (-3.82, -1.01)
WK_F	-4.307	0.000	-3.02 (-4.42, -1.62)
WK_S	-2.448	0.015	-1.71 (-3.11, -0.31)
TS	-1.792	0.073	-1.51 (-3.19, 0.17)
GR	-0.307	0.759	-0.10 (-0.79, 0.58)
NG	-0.762	0.446	-0.14 (-0.51, 0.23)
GR_{m3}	4.928	0.000	2.39 (1.42, 3.36)
NG_{m3}	1.183	0.237	0.25 (-0.17, 0.67)
GR:TS	-0.137	0.891	-0.20 (-3.08, 2.68)
NG:TS	2.409	0.016	2.56 (0.43, 4.68)
GR_{m3}:TS	2.920	0.004	4.58 (1.44, 7.71)
NG_{m3}:TS	-0.078	0.938	-0.07 (-1.88, 1.74)
	F value	$\Pr(> F)$	EDF
yday	10.607	0.000	6.607
RH_{rl}	4.979	0.000	5.330
RH_{dv}	0.582	0.013	0.878
PR	9.097	0.000	7.687
EW	0.000	0.996	0.000
NS	0.691	0.114	3.558
TM_{rl}	3.311	0.000	4.247
TM_{dv}	1.088	0.003	2.215
