# Supplementary material for: Seasonal asthma in Melbourne, Australia, and some observations on the occurrence of thunderstorm asthma and its predictability
Source: PLoS One. 2018 Apr 12;13(4):e0194929. doi: 10.1371/journal.pone.0194929 (PMC5896915; doi:10.1371/journal.pone.0194929)
Supplement: S8 Table — Summary of the fit for model 5 (see S3 Table). See the caption of S4 Table for further details. (PDF) [file pone.0194929.s027.pdf]

	t value	$\Pr(>\|t\|)$	Effect size
(Intercept)	57.306	0.000	19.49 (18.81, 20.17)
TS	3.243	0.001	1.51 (0.58, 2.44)
WK_M	1.279	0.201	0.61 (-0.35, 1.57)
WK_{Tu}	-2.628	0.009	-1.27 (-2.23, -0.30)
WK_{We}	-4.582	0.000	-2.21 (-3.17, -1.24)
WK_{Th}	-5.847	0.000	-2.83 (-3.80, -1.86)
WK_F	-6.844	0.000	-3.30 (-4.26, -2.34)
WK_S	-6.074	0.000	-2.87 (-3.81, -1.92)
	F value	$\Pr(> F)$	EDF
yday	72.610	0.000	7.972
RH_{rl}	5.915	0.000	5.244
RH_{dv}	0.614	0.071	2.528
PR	0.858	0.004	1.444
EW	0.000	0.808	0.000
NS	0.020	0.288	0.161
TM_{rl}	9.038	0.000	6.805
TM_{dv}	1.866	0.000	3.181
O_3	0.340	0.145	1.730
$PM_{2.5}$	1.364	0.009	4.173
