# Supplementary material for: Seasonal asthma in Melbourne, Australia, and some observations on the occurrence of thunderstorm asthma and its predictability
Source: PLoS One. 2018 Apr 12;13(4):e0194929. doi: 10.1371/journal.pone.0194929 (PMC5896915; doi:10.1371/journal.pone.0194929)
Supplement: S9 Table — Summary of the fit for model 6 (see S3 Table). See the caption of S4 Table for further details. (PDF) [file pone.0194929.s028.pdf]

	t value	$\Pr(>\|t\|)$	Effect size
(Intercept)	23.226	0.000	18.43 (16.84, 20.02)
TS	2.386	0.017	2.11 (0.34, 3.89)
WK_M	0.189	0.850	0.21 (-1.96, 2.37)
WK_{Tu}	-0.632	0.527	-0.70 (-2.89, 1.50)
WK_{We}	-2.103	0.036	-2.29 (-4.46, -0.11)
WK_{Th}	-1.740	0.082	-1.93 (-4.14, 0.29)
WK_F	-2.891	0.004	-3.21 (-5.43, -0.99)
WK_S	-1.506	0.133	-1.65 (-3.85, 0.54)
	F value	$\Pr(> F)$	EDF
yday	7.173	0.000	2.680
RH_{rl}	4.452	0.000	5.999
RH_{dv}	1.739	0.001	3.713
PR	5.988	0.000	7.039
EW	0.000	0.491	0.000
NS	0.129	0.176	0.656
TM_{rl}	3.327	0.000	3.931
TM_{dv}	1.913	0.000	3.663
O_3	0.000	0.545	0.000
$PM_{2.5}$	0.000	0.496	0.000
GR	0.000	0.289	0.000
NG	0.642	0.018	1.593
