# Supplementary material for: Seasonal asthma in Melbourne, Australia, and some observations on the occurrence of thunderstorm asthma and its predictability
Source: PLoS One. 2018 Apr 12;13(4):e0194929. doi: 10.1371/journal.pone.0194929 (PMC5896915; doi:10.1371/journal.pone.0194929)
Supplement: S10 Table — Summary of the fit for model 7 (see S3 Table). See the caption of S4 Table for further details. (PDF) [file pone.0194929.s029.pdf]

	t value	$\Pr(>\|t\|)$	Effect size
(Intercept)	24.076	0.000	18.45 (16.91, 19.98)
TS	2.603	0.009	2.20 (0.51, 3.89)
WK_M	0.300	0.765	0.31 (-1.77, 2.39)
WK_{Tu}	-0.740	0.460	-0.79 (-2.91, 1.34)
WK_{We}	-2.116	0.035	-2.21 (-4.30, -0.12)
WK_{Th}	-1.740	0.082	-1.86 (-4.00, 0.28)
WK_F	-3.057	0.002	-3.28 (-5.43, -1.14)
WK_S	-1.852	0.064	-1.95 (-4.05, 0.16)
	F value	$\Pr(> F)$	EDF
yday	5.618	0.000	2.571
RH_{rl}	4.109	0.000	6.661
RH_{dv}	1.778	0.001	3.721
PR	3.940	0.000	6.298
EW	0.000	0.666	0.000
NS	0.215	0.096	0.729
TM_{rl}	1.701	0.000	2.577
TM_{dv}	2.270	0.000	3.490
O_3	0.121	0.142	0.579
$PM_{2.5}$	0.219	0.198	1.305
GR	0.000	0.784	0.000
NG	0.044	0.246	0.299
GR_{m3}	3.925	0.000	3.752
NG_{m3}	4.170	0.000	5.599
