# Supplementary material for: Seasonal asthma in Melbourne, Australia, and some observations on the occurrence of thunderstorm asthma and its predictability
Source: PLoS One. 2018 Apr 12;13(4):e0194929. doi: 10.1371/journal.pone.0194929 (PMC5896915; doi:10.1371/journal.pone.0194929)
Supplement: S11 Table — Summary of the fit for model 8 (see S3 Table). See the caption of S4 Table for further details. (PDF) [file pone.0194929.s030.pdf]

	t value	$\Pr(>\|t\|)$	Effect size
(Intercept)	19.712	0.000	17.16 (15.42, 18.90)
WK_M	0.135	0.893	0.14 (-1.98, 2.27)
WK_{Tu}	-0.841	0.401	-0.92 (-3.10, 1.26)
WK_{We}	-2.238	0.026	-2.40 (-4.55, -0.25)
WK_{Th}	-1.770	0.077	-1.93 (-4.12, 0.25)
WK_F	-2.964	0.003	-3.26 (-5.45, -1.06)
WK_S	-1.518	0.129	-1.63 (-3.77, 0.52)
TS	-1.087	0.277	-1.35 (-3.84, 1.13)
GR	-0.333	0.739	-0.16 (-1.15, 0.82)
NG	-0.717	0.473	-0.16 (-0.60, 0.28)
GR_{m3}	3.715	0.000	2.57 (1.19, 3.95)
NG_{m3}	1.091	0.276	0.29 (-0.24, 0.81)
GR:TS	-0.710	0.478	-1.77 (-6.77, 3.22)
NG:TS	1.334	0.183	2.02 (-1.01, 5.04)
GR_{m3}:TS	2.479	0.013	7.02 (1.36, 12.69)
NG_{m3}:TS	0.130	0.896	0.16 (-2.28, 2.60)
	F value	$\Pr(> F)$	EDF
yday	5.257	0.000	2.652
RH_{rl}	4.558	0.000	6.678
RH_{dv}	1.400	0.004	3.649
PR	5.355	0.000	6.950
EW	0.000	0.747	0.000
NS	0.243	0.079	0.733
TM_{rl}	3.638	0.000	6.139
TM_{dv}	2.160	0.000	3.509
O_3	0.173	0.117	0.662
$PM_{2.5}$	0.176	0.228	1.132
